# Aberrant extracellular dopamine clearance in the prefrontal cortex exhibits ADHD‐like behavior in NCX3 heterozygous mice

**DOI:** 10.1111/febs.17339

**Published:** 2024-12-03

**Authors:** Ryo Inagaki, Satomi Kita, Nozomu Niwa, Kohji Fukunaga, Takahiro Iwamoto, Shigeki Moriguchi

**Affiliations:** ^1^ Research Center for Pharmaceutical Development, Graduate School of Pharmaceutical Sciences Tohoku University Sendai Japan; ^2^ Department of Pharmacology, Faculty of Pharmaceutical Sciences Tokushima Bunri University Japan; ^3^ Department of Pharmacology, Graduate School of Pharmaceutical Sciences Tohoku University Sendai Japan; ^4^ Department of Pharmacology, Faculty of Medicine Fukuoka University Japan

**Keywords:** ADHD, CaMKII, DAT, dopamine, NCX3

## Abstract

Attention‐deficit/hyperactivity disorder (ADHD) is a neurodevelopmental disorder that involves dopaminergic dysfunction in the prefrontal cortex (PFC), manifesting hyperactivity, inattention, and cognitive deficits. However, the ADHD‐associated candidate genes underlying dopaminergic neurotransmission alterations remain poorly defined. Here, we identified the abundant localization of sodium‐calcium exchanger 3 (NCX3) levels in the dopaminergic neurons of the ventral tegmental area, a major source of dopaminergic innervation to the PFC. We confirmed that NCX3 knockdown in N27 cells caused aberrant dopamine influx through the strong interaction between calcium/calmodulin‐dependent protein kinase II alpha and dopamine transporter. In addition, we assessed behavioral changes and underlying molecular properties in NCX3 heterozygous (NCX3^+/−^) mice. NCX3^+/−^ mice exhibited hyperactivity, cognitive deficits, and social dysfunction which were alleviated by treating with methylphenidate. Furthermore, NCX3^+/−^ mice displayed a persistent elevation of basal dopamine levels and decreased extracellular levels of dopamine triggered by social stimuli in the PFC of NCX3^+/−^ mice. In agreement with the rise in extracellular dopamine levels in the PFC, NCX3^+/−^ mice showed activation of dopamine D1 receptor signaling pathways in the PFC compared to wild‐type mice. Thus, deficiency of NCX3 leads to impaired dopaminergic neurotransmission in the PFC, which likely accounts for the ADHD‐like behavior in NCX3^+/−^ mice.

## Graphical abstract

NCX3 knockdown in dopaminergic neurons of the VTA induces a direct physical interaction between phospho‐CaMKII and DAT, inducing excess extracellular dopamine levels due to the disruption of dopamine clearance in the PFC. In concordance with the increase of extracellular dopamine levels in the PFC, NCX3^+/−^ mice exhibited the activation of dopamine D1 receptor signaling pathways, which promote hyperactivity, cognitive deficit, and social dysfunction associated with ADHD.

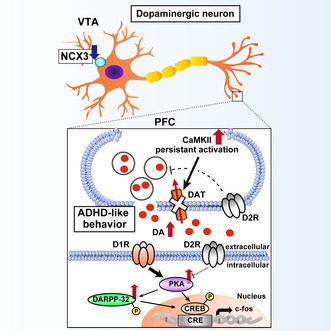

**Abbreviations**
AC: adenylate cyclase
ADHD: attention‐deficit/hyperactivity disorder
AMPAR: α‐amino‐3‐hydroxy‐5‐methyl‐4‐isoxazolepropionic acid receptor
CaMKII: calcium/calmodulin‐dependent protein kinase II
cAMP: cyclic adenosine monophosphate
CyC: cytochrome C
D1+: dopamine D1‐positive neurons
D1R: dopamine D1 receptor
D2+: dopamine D2‐positive neurons
D2R: dopamine D2 receptor
DA: dopamine
DAPI: 4′,6‐diamidino‐2‐phenylindole
DAT: dopamine transporter
fEPSPs: field excitatory postsynaptic potentials
GAPDH: glyceraldehyde 3‐phosphate dehydrogenase
HFS: high‐frequency stimulation
IB: immunoblot
IP: immunoprecipitation
LTP: long‐term potentiation
MP: methylphenidate
mPFC: medial PFC
NCX: sodium‐calcium exchanger
NMDAR: *N*‐methyl‐d‐aspartate receptor
nNOS: neuronal nitric oxide synthase
PBP: parabrachial pigmented nucleus
PBS: phosphate‐buffered saline
PFC: prefrontal cortex
PKA: protein kinase A
qRT‐PCR: quantitative reverse transcription polymerase chain reaction IHC immunohistochemistry
SEM: standard error of the mean
SNc: substantia nigra pars compacta
STR: striatum
TH: tyrosine hydroxylase
*V*_max_
: the maximal reaction velocity
VTA: ventral tegmental area
WT: wild‐type

## Introduction

Attention‐deficit/hyperactivity disorder (ADHD) is a neurodevelopmental disorder affecting approximately 5% of children and 2.5% of adults [1]. It is characterized by hyperactivity, inattention, impulsivity, antisocial, and cognitive impairments [1].

Midbrain dopaminergic neurons serve as the principal source of dopamine innervation throughout the various brain regions, being primarily localized within the ventral tegmental area (VTA) and substantia nigra pars compacta (SNc). The neurons within the VTA project their axons and replicate toward the cortical limbic area and play a fundamental role in regulating emotional behavior. On the other hand, dopaminergic neurons from the SNc innervate toward the dorsal/lateral striatum, areas associated with motor functions [2].

Patients with underlying defects in the neuronal connections of specific brain nuclei related to ADHD symptoms structural and functional abnormalities of the prefrontal cortex (PFC) were observed in the brains of patients during brain imaging analyses. For instance, positron emission tomography imaging studies have demonstrated reduced optimal levels of catecholamine (e.g., dopamine, norepinephrine) input to the PFC in patients with ADHD, which correspond with the severity of ADHD symptom [3, 4]. These data provide convincing evidence that most medications are used to treat ADHD by the enhancement of dopamine and norepinephrine neurotransmission. In addition, genetic studies have identified a variety of genes related to dopamine (e.g., dopamine transporter [DAT] and DRD4) and norepinephrine (e.g., DBH and DRD4) that are involved in the pathogenesis of ADHD [5]. Aberrant catecholamine levels within the PFC have been implicated as a crucial factor in ADHD‐related pathophysiological mechanisms.

The Na^+^/Ca^2+^ exchanger (NCX) is an antiporter protein predominantly localized in neuronal plasma membranes that regulates intracellular Ca^2+^ concentration through exchange with three Na^+^ ions, either in one Ca^2+^ efflux (forward mode) or an influx (reverse mode), depending on the Na^+^ gradient [6, 7]. The NCX family is mainly comprised of three genes coding for three different proteins NCX1–3, and each isoform carries various splice variants that are expressed in specific tissues, which in turn exhibit distinct cellular mechanisms [8, 9]. Previous studies have reported NCX exchangers to be associated with brain diseases, such as cerebral ischemia, Alzheimer's disease, and Parkinson's disease [10, 11, 12, 13]. Under these circumstances, SLC8A3, the gene encoding NCX3, was recently identified as a candidate gene related to ADHD [14]. Regarding psychiatric disorders such as ADHD, the role of NCX3 in both pathological conditions and its contribution to pathogenesis remains unclear.

The DAT is the integral plasma membrane protein that regulates the spatial and temporal dynamics of dopaminergic neurotransmission by the reuptake of dopamine into the cytosol from the synaptic cleft. DAT is specifically expressed in the presynaptic region of dopaminergic neurons and has been the focus of numerous studies on dopamine‐related diseases, including ADHD, not only because of its vital role in dopaminergic neurotransmission but also because the blockage of DAT serves as a therapeutic target for ADHD. Additionally, ADHD appears to have highly heritable components, and several rare coding variants of hDAT have been identified in patients with ADHD, including V24M, L167P, A559V, and R615C [15, 16, 17, 18]. A recent study also demonstrated that the hDAT A559V variant elicits an anomalous DAT‐mediated dopamine efflux similar to that caused by amphetamine‐like psychostimulants, and that potentially, calcium/calmodulin‐dependent protein kinase II (CaMKII) could modulate similar anomalous DA efflux [19, 20].

In the present study, we investigate both hyperlocomotion and cognitive dysfunction observed in NCX3^+/−^ mice caused by the persistent activation of CaMKIIα and its aberrant interaction with DAT. Thus, NCX3 deficiency‐induced disruption of dopaminergic neurotransmission in the PFC could be a crucial aspect for understanding the onset of ADHD pathology [5, 14].

## Results

### NCX3 is abundantly localized in dopaminergic neurons of the ventral tegmental area

**Fig. 1:**
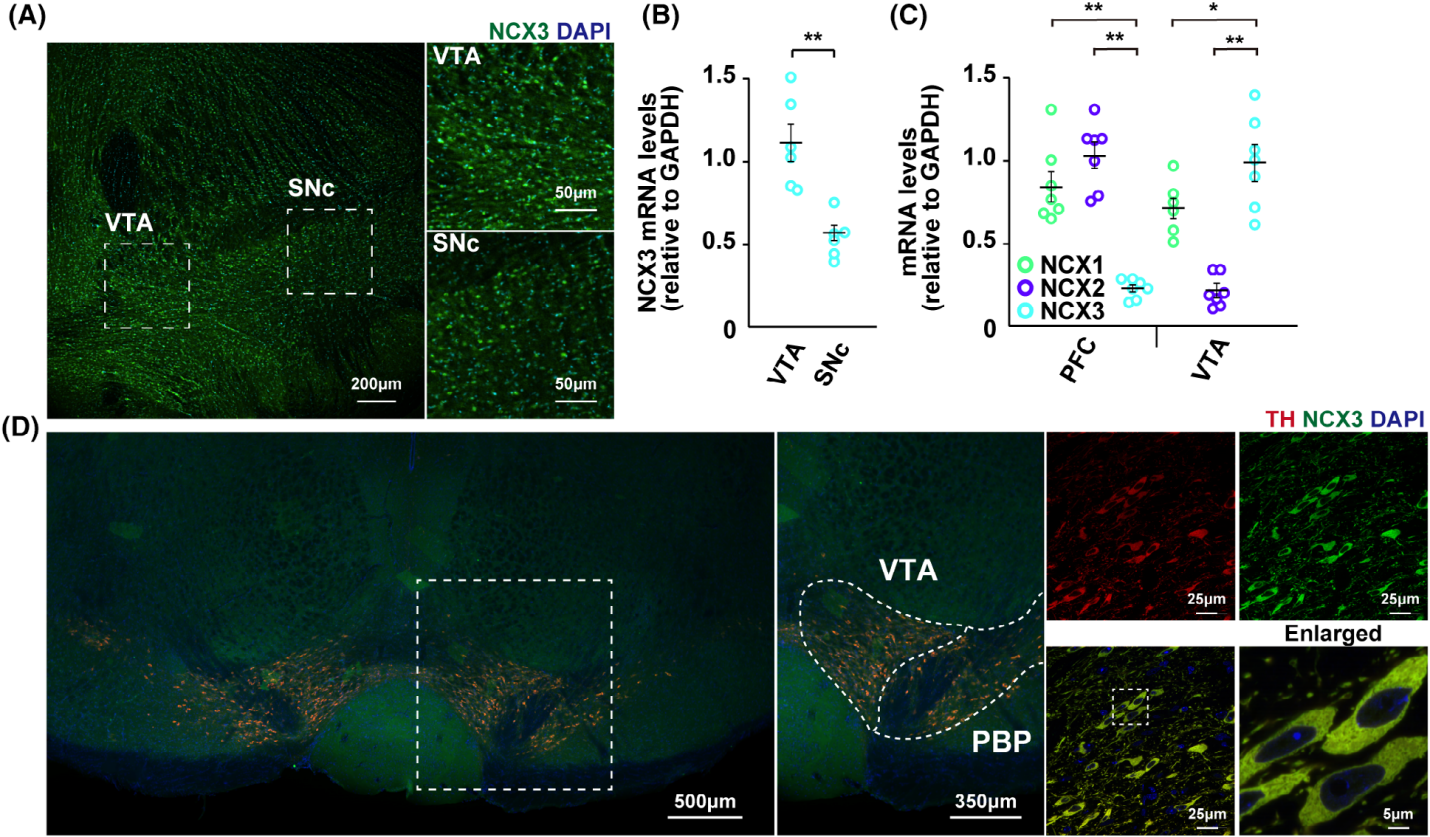
Localization of NCX3 in dopaminergic neurons of the ventral tegmental area. (A) Representative images of coronal midbrain sections containing the VTA and SNc stained with an anti‐NCX3 antibody (green). Scale bar: 200 μm at low magnification and 50 μm at enlarged images (VTA and SNr regions). Representative images are shown from three independent experiments (*n* = 3). (B) Quantitative analysis of NCX3 mRNA in the VTA and the SNc regions. The analysis revealed increased expression of NCX3 mRNA in the VTA compared to the SNc (*n* = 6 each, *t*‐test, **P < 0.01). (C) Quantitative analysis of NCX1–3 mRNA in the PFC and VTA regions. The analysis revealed that NCX3 mRNA levels were significantly higher compared with the levels of other NCX isoforms in the VTA. In contrast, NCX3 mRNA levels were significantly lower than the levels of other NCX isoforms in the PFC (*n* = 7 each, *t*‐test, **P < 0.01, *P < 0.05). (D) Confocal microscopy images showing double staining of VTA slices for NCX3 (green) and TH (red), and merged images at multiple magnifications. Data are expressed as the means ± standard error of the mean (SEM). Scale bars, 500 μm at low magnification (midbrain region); 25 μm in middle magnification (VTA and SNr regions); 25 and 5 μm in high magnification (VTA region). Representative images are shown from three independent experiments (*n* = 3). DAPI, 4′,6‐diamidino‐2‐phenylindole; GAPDH, glyceraldehyde 3‐phosphate dehydrogenase; NCX, sodium‐calcium exchanger; PBP, parabrachial pigmented nucleus; SNc, substantia nigra pars compacta; VTA, ventral tegmental area.

Immunohistochemistry analysis revealed abundant subpopulations of NCX3 in both the VTA and SNc regions of the midbrain, with notably stronger staining intensity observed in the VTA relative to the SNc (Fig. 1A). Consistent with immunohistochemical findings, an upregulation of NCX3 mRNA was detected in the VTA compared to the SNc (VTA: 1.11 ± 0.12, SNc: 0.53 ± 0.05 mRNA levels relative to GAPDH, *n* = 6 each) (Fig. 1B). Notably, NCX3 mRNA levels were significantly higher than the levels of other NCX isoforms in VTA, whereas NCX3 mRNA levels were significantly lower than the levels of other NCX isoforms in the PFC, the region to which the VTA dopamine neuron is primarily projected ([PFC] NCX1; 0.84 ± 0.09; NCX2; 1.04 ± 0.08; NCX3; 0.21 ± 0.02 [VTA] NCX1; 0.71 ± 0.07; NCX2; 0.21 ± 0.03; NCX3; 0.99 ± 0.10 mRNA levels relative to GAPDH, *n* = 7) (Fig. 1C). Furthermore, NCX3 expression was identified in TH‐positive neurons in the VTA (Fig. 1D).

### NCX3 knockdown decreased dopamine intake due to aberrant functional coupling between DAT and CaMKII

**Fig. 2:**
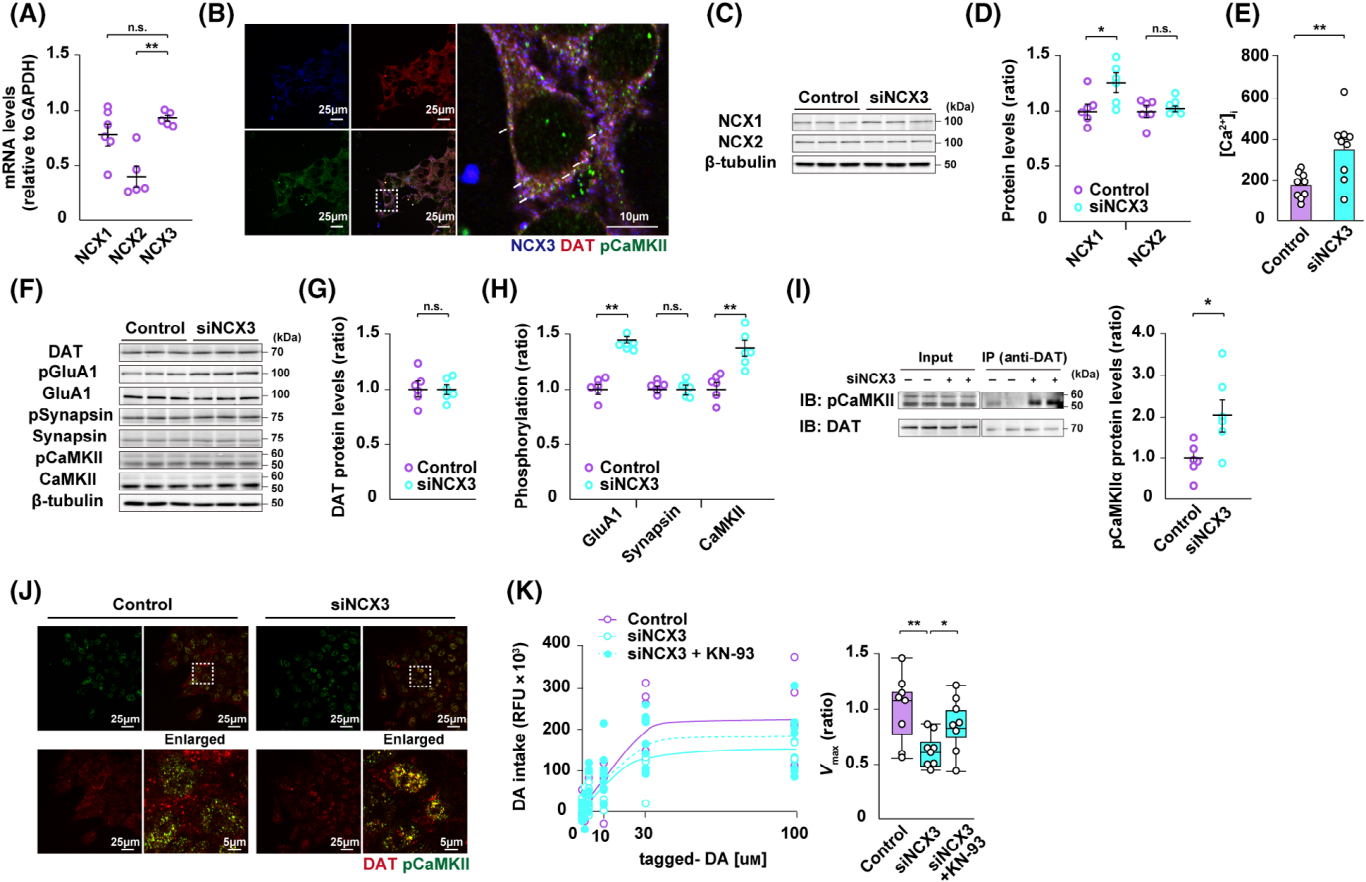
NCX3 knockdown induces aberrant dopamine intake through changes in the physical and functional coupling between DAT and CaMKII. (A) Quantitative analysis of NCX1–3 mRNA in the N27 dopaminergic cells. The analysis revealed that NCX3 mRNA levels were significantly higher in N27 dopaminergic neurons compared with the levels of other NCX isoforms (*n* = 5–6 each, *t*‐test, **P < 0.01). (B) Confocal microscopy images showing triple staining of N27 cells for autophosphorylated CaMKIIα (Thr‐286) (green), DAT (red), NCX3 (blue), and merged images. White arrows indicate the colocalization between phospho‐CaMKIIα, DAT, and NCX3. Scale bars: 25 and 10 μm (enlarged) in the images. (C, D) Representative immunoblots of lysates from N27 cells probed with antibodies recognizing NCX1, NCX2, and β‐tubulin. Quantitative analyses from obtained blotting data revealed that NCX1 protein levels were significantly increased in siNCX3 N27 cells (*n* = 6 per group, *t*‐test, *P < 0.05). (E) Quantification of [Ca^2+^]_i_ in the control and siNCX3 N27 cells. The silencing of NCX3 caused an increase in basal [Ca^2+^]_i_ (*n* = 9 per group, *P < 0.05, inter‐group comparison). (F) Representative immunoblots of lysates from N27 cells probed with antibodies recognizing DAT, phosphorylated GluA1 (Ser‐831), GluA1, autophosphorylated CaMKIIα (Thr‐286), CaMKII, phosphorylated Synapsin I (Ser‐603), Synapsin I and β‐tubulin. Representative images are shown from two independent experiments (*n* = 2). (G) Quantitative analyses of DAT protein levels are presented in (G). (H) Quantitative analysis of phosphorylation, as shown in (H). CaMKIIα (Thr‐286) autophosphorylation and its downstream substrates GluA1 phosphorylation were significantly increased in siNCX3 N27 cells (*n* = 6 per group, *t*‐test, **P < 0.01). (I) Representative immunoprecipitation and whole‐cell lysate input for autophosphorylated CaMKIIα (Thr‐286), along with grouped analysis of autophosphorylated CaMKIIα (Thr‐286)–DAT interaction. NCX3 knockdown promoted strong physical interaction between phospho‐CaMKIIα and DAT (*n* = 6 per group, *t*‐test, *P < 0.05). (J) Confocal microscopy images showing double staining of N27 cells for autophosphorylated CaMKIIα (Thr‐286) (green) and DAT (red) and merged images. NCX3 knockdown promoted strong colocalization between phospho‐CaMKIIα and DAT. Scale bars: 25 and 5 μm (enlarged) in the images. (K) Tagged DA uptake kinetics in control or siNCX3 N27 cells treated either with vehicle or KN‐93. siNCX3 cells showed suppressed dopamine intake compared to control cells, whereas CaMKII inhibition by KN‐93 partially rescued siNCX3‐induced disruption of dopamine intake (*n* = 8 per group, *F*_(2,21)_ = 4.46, **P < 0.01, and *P < 0.05, inter‐group comparison). Data are expressed as the means ± standard error of the mean (SEM). CAMKII, calcium/calmodulin‐dependent protein kinase II; DA, dopamine; DAT, dopamine transporter; IB, immunoblot; IP, immunoprecipitation; NCX, sodium‐calcium exchanger.

First, we quantitatively analyzed NCX isoform mRNA in the N27 dopaminergic cells. We found that NCX3 mRNA levels were significantly higher in N27 dopaminergic neuron compared with the levels of other NCX isoforms (NCX1; 0.78 ± 0.09; NCX2; 0.41 ± 0.09; NCX3; 0.94 ± 0.02, *n* = 5–6 each) (Fig. 2A). Given NCX3's involvement in intracellular calcium homeostasis and dopamine clearance, we detected the colocalization of NCX3 with Dopamine transporter and autophosphorylated CaMKII in N27 dopaminergic neurons (Fig. 2B). Subsequently, we transfected N27 dopaminergic neurons with siNCX3 or a mock (control) to investigate the role of NCX3 in the regulation of dopaminergic neurons. The silencing of NCX3 caused an increase in basal [Ca^2+^]_i_ (Control; 169.80 ± 20.56 nm; siNCX3; 343.24 ± 50.60 nm, *n* = 9 each) (Fig. 2E). Consistent with the results of basal [Ca^2+^]_i_, siNCX3 cells showed a significant increase in autophosphorylated CaMKII at the Thr‐286 residue, and its downstream substrates, specifically GluA1 phosphorylation at Ser‐831, compared to control cells ([siNCX3] CaMKIIα (Thr‐286): 1.35 ± 0.07; GluA1 (Ser‐831): 1.43 ± 0.03 ratio relative to control, *n* = 6 each) (Fig. 2F,H). Meanwhile, no significant difference was observed in DAT expression and Synapsin I phosphorylation at the Ser‐603 residue between mock and siNCX3 cells (Fig. 2F–H). It is noteworthy that the silencing of NCX3 resulted in an increase in NCX1 mRNA as a compensatory mechanism to counteract reduced NCX3 mRNA, however, increased NCX1 mRNA does not appear to contribute to normalization of intracellular Ca^2+^ concentrations and intracellular calcium signaling pathway ([NCX1 expression] siNCX3; 1.26 ± 0.8 ratio relative to control, *n* = 6 each) (Fig. 2C,D).

Previous studies have highlighted CaMKII as a functional substrate of DAT, suggesting that aberrant CaMKII activity induced by siNCX3 might alter the physical interaction between DAT and CaMKII [21]. Therefore, we evaluated both protein–protein interaction and colocalization between phospho‐CaMKIIα and DAT through co‐immunoprecipitation and immunohistochemistry analysis, respectively. As depicted in Fig. 2I,J, NCX3 siNCX3 knockdown significantly enhanced the physical interaction between phospho‐CaMKIIα and DAT ([siNCX3] CaMKIIα Thr‐286: 2.07 ± 0.38 ratio relative to control, *n* = 6 each, Fig. 2I).

Consistent with the findings from co‐immunoprecipitation and immunohistochemistry analyses, siNCX3‐treated cells exhibited disrupted dopamine uptake relative to control cells. Moreover, treatment with KN‐93 (Merck Millipore, Burlington, MA, USA) at 5 μm, an inhibitor of CaMKIIα, partially ameliorated siNCX3‐induced dysregulated dopamine uptake (siNCX3; 0.63 ± 0.06, siNCX3 plus KN‐93; 0.86 ± 0.10 *V*_max_ ratio relative to control, *n* = 8 each) (Fig. 2K).

### Methylphenidate treatment ameliorated both hyperactivity and cognitive deficits in NCX3 heterozygous mice

To investigate the potential pathological relevance of reduced NCX3 underlying dopaminergic dysfunction, we conducted several behavioral tasks associated with locomotion, motor coordination, sociality, and cognition in NCX3 heterozygous (NCX3^+/−^) mice relative to wild‐type (WT) mice.

**Fig. 3:**
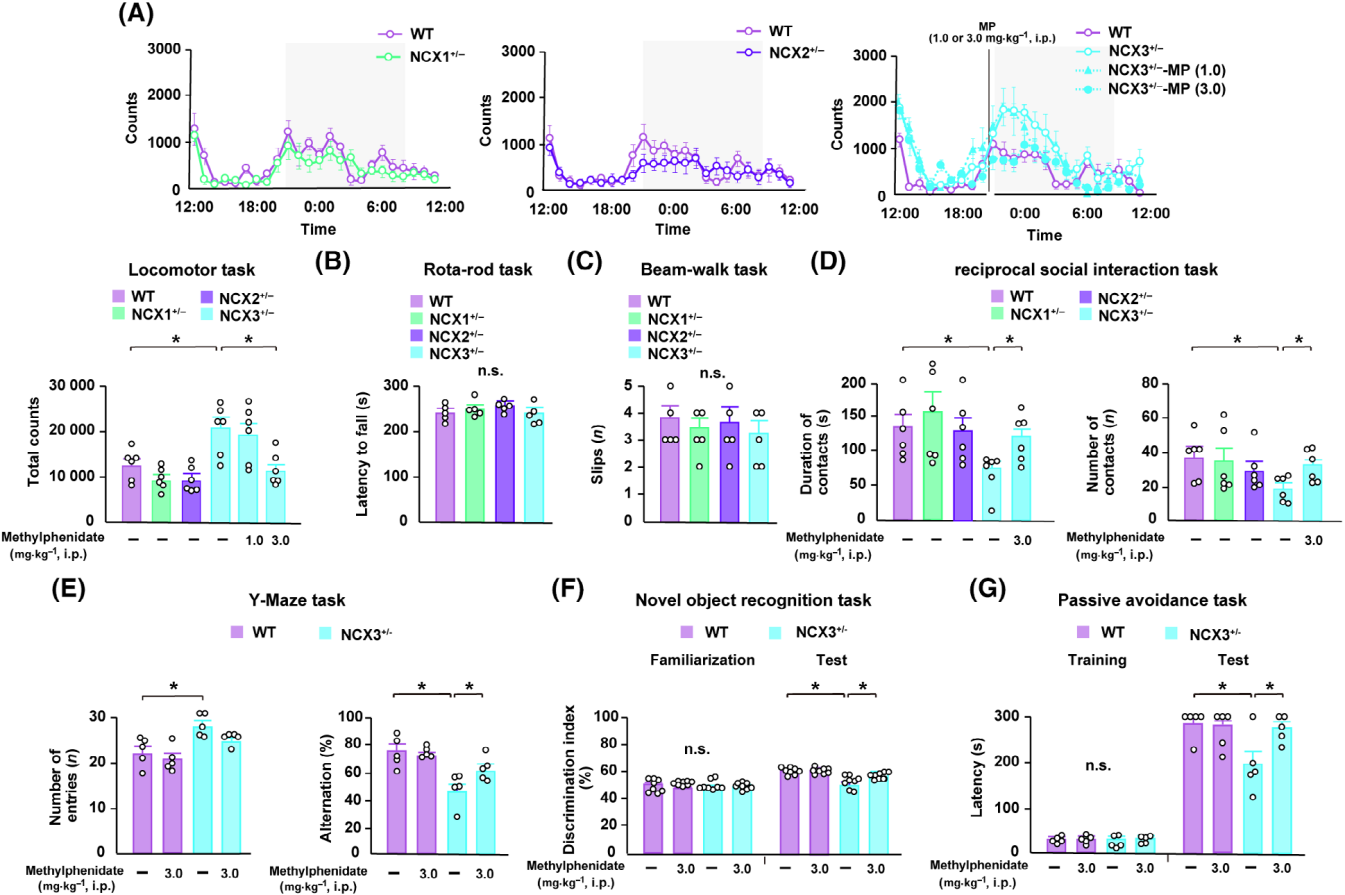
NCX3^+/−^ mice show hyperactivity, social dysfunction, and cognitive impairment which are ameliorated by methylphenidate. (A) Spontaneous locomotor activity tests. The total locomotor activity over the 24 h periods was significantly increased in NCX3^+/−^ mice relative to that in WT, NCX1^+/−^, and NCX2^+/−^ mice. Acute treatment with methylphenidate at 3.0 mg·kg^−1^ i.p. significantly ameliorated the hyperactivity observed in NCX3^+/−^ mice (*n* = 6 per group, *F*_(5,30)_ = 7.68, *P < 0.05, inter‐group comparison). The gray box in the upper figures indicates the 12 h period when the lights were turned off (9 p.m. to 9 a.m). (B, C) Motor function tasks. No significant differences were observed between the WT and NCX1–3^+/−^ mice groups in the rotarod task (B) or beam‐walk task (C). (D) Reciprocal social interaction task. NCX3^+/−^ mice exhibited a significantly reduced number and latency of social interactions with novel unfamiliar mice compared to WT mice. Abnormal social recognition shown in NCX3^+/−^ mice was significantly ameliorated by methylphenidate (*n* = 6 per group, [Duration] *F*_(4,25)_ = 2.42, *P < 0.05, inter‐group comparison, [Number] *F*_(4,25)_ = 1.85, *P < 0.05, inter‐group comparison). (E–G) Memory‐related behavioral tasks. The total number of entries or alternations in the Y‐maze task was measured in WT, NCX3^+/−^, and methylphenidate‐treated NCX3^+/−^ mice (E). The total number of entries in the Y‐maze task was significantly increased in NCX3^+/−^ mice relative to WT mice, while the alternations of NCX3^+/−^ mice decreased relative to WT mice. Single treatment of methylphenidate at 3.0 mg kg^−1^ i.p. significantly rescued abnormal locomotors and alternations seen in NCX3^+/−^ mice (*n* = 5 per group, [Number] *F*_(3,16)_ = 7.34, *P < 0.05, inter‐group comparison, [Alternations] *F*_(3,16)_ = 9.51, *P < 0.05, inter‐group comparison). The number of times a mouse recognized a novel object was measured in WT, NCX3^+/−^, and methylphenidate‐treated NCX3^+/−^ mice (F). The number of times a mouse recognized a novel object in the test trial significantly decreased in NCX3^+/−^ mice relative to WT mice, and methylphenidate treatment rescued the test outcomes (*n* = 8 per group, *F*_(3,28)_ = 14.54, *P < 0.05, inter‐group comparison). Latency time in the training and test trials of the passive avoidance task (G). Latency time in test trials in NCX3^+/−^ mice significantly decreased relative to that in WT mice, and methylphenidate treatment rescued the decline in memory retention (*n* = 5 per group, *F*_(3,16)_ = 8.26, *P < 0.05, inter‐group comparison). Data are expressed as the means ± standard error of the mean (SEM). MP, methylphenidate; NCX, sodium‐calcium exchanger; WT, wild‐type.

In the spontaneous locomotor activity task, total locomotor activity significantly increased in NCX3^+/−^ mice compared with that in WT mice (WT: 12625.33 ± 1380.80, NCX3^+/−^: 20388.88 ± 2322.87, *n* = 6 each) (see Fig. 3A). Notably, acute treatment with methylphenidate at 3.0 mg·kg^−1^ i.p. significantly ameliorated the heightened locomotor activity observed in NCX3^+/−^ mice (11485.17 ± 1449.01, *n* = 6 each). In contrast, NCX1 and NCX2 heterozygous (NCX1^+/−^, NCX2^+/−^) mice showed no change in locomotor activity compared with WT mice (Fig. 3A).

Next, we assessed motor coordination, which is related to striatal dopamine function using the rotarod and beam‐walk tasks [22]. We confirmed that no abnormalities were observed in NCX1–3^+/−^ mice relative to WT mice during the rotarod and beam‐walk tasks (Fig. 3B,C).

We then evaluated social abnormalities using reciprocal social interaction tasks. We found reduced numbers and latencies of social interactions with novel unfamiliar mice in NCX3^+/−^ mice relative to WT mice, without changes observed in NCX1^+/−^ and NCX2^+/−^ mice (WT: latency; 134.00 ± 18.03 s, number; 36.67 ± 5.31, NCX3^+/−^: latency; 73.17 ± 11.32 s, number; 18.17 ± 2.85, *n* = 6 each) (Fig. 3D). Furthermore, abnormal social recognition was significantly ameliorated treated by methylphenidate in NCX3^+/−^ mice (3.0 mg·kg^−1^: latency; 120.83 ± 13.43 s, number; 30.83 ± 3.80, *n* = 6 each) (Fig. 3D).

We previously reported disrupted memory formation and retention in NCX3^+/−^ mice [11]. Hence, we examined whether treatment with methylphenidate could improve cognitive deficits in NCX3^+/−^ mice using memory‐related behavioral tasks involving the Y‐maze task, novel object recognition task, and passive avoidance task (Fig. 3E–G). In the Y‐maze task, the number of arm entries was significantly increased in NCX3^+/−^ mice, and treatment of methylphenidate partly this effect, although not significantly (WT: 22.00 ± 1.41, NCX3^+/−^: 28.00 ± 1.26, NCX3^+/−^ plus methylphenidate: 24.4 ± 0.81, *n* = 6 each) (Fig. 3E). Additionally, the percentage of alternations also significantly decreased in NCX3^+/−^ mice, and treatment with methylphenidate significantly ameliorated this deficit (WT: 74.00 ± 4.84%, NCX3^+/−^: 46.56 ± 5.22%, NCX3^+/−^ plus methylphenidate: 61.98 ± 3.71%, *n* = 6 each) (Fig. 3E). Consistent with previous reports [11], NCX3^+/−^ mice failed to discriminate between familiar and novel objects in the novel object recognition task, and this was significantly ameliorated by methylphenidate treatment (WT: 60.62 ± 0.83%, NCX3^+/−^: 51.88 ± 1.58%, NCX3^+/−^ plus methylphenidate: 56.52 ± 0.68%, *n* = 6 each) (Fig. 3F) [11]. In the step‐through passive avoidance task, no differences in the latency to enter the dark compartment between the groups during the training trial were found (Fig. 3G). However, NCX3^+/−^ mice exhibited significantly reduced latency to remain in the light compartment during the retention trial relative to WT mice (WT: 295.20 ± 4.80 s, NCX3^+/−^: 196.80 ± 28.60 s, *n* = 6 each) (Fig. 3G). Meanwhile, treatment of methylphenidate significantly improved the latency time into the light compartment in NCX3^+/−^ mice (NCX3^+/−^ plus methylphenidate: 275.40 ± 11.87 s, *n* = 6 each) (Fig. 3G).

### Aberrant physical interaction between DAT and CaMKIIα observed in the PFC of NCX3 heterozygous mice

To address the behavioral phenotypes characterized by both hyperactivity and cognitive deficits in NCX3^+/−^ mice, we investigated the molecular properties underlying dopaminergic dysfunction in the PFC and dorsal striatum (STR) extracts obtained with or without methylphenidate treatment in NCX3^+/−^ mice.

**Fig. 4:**
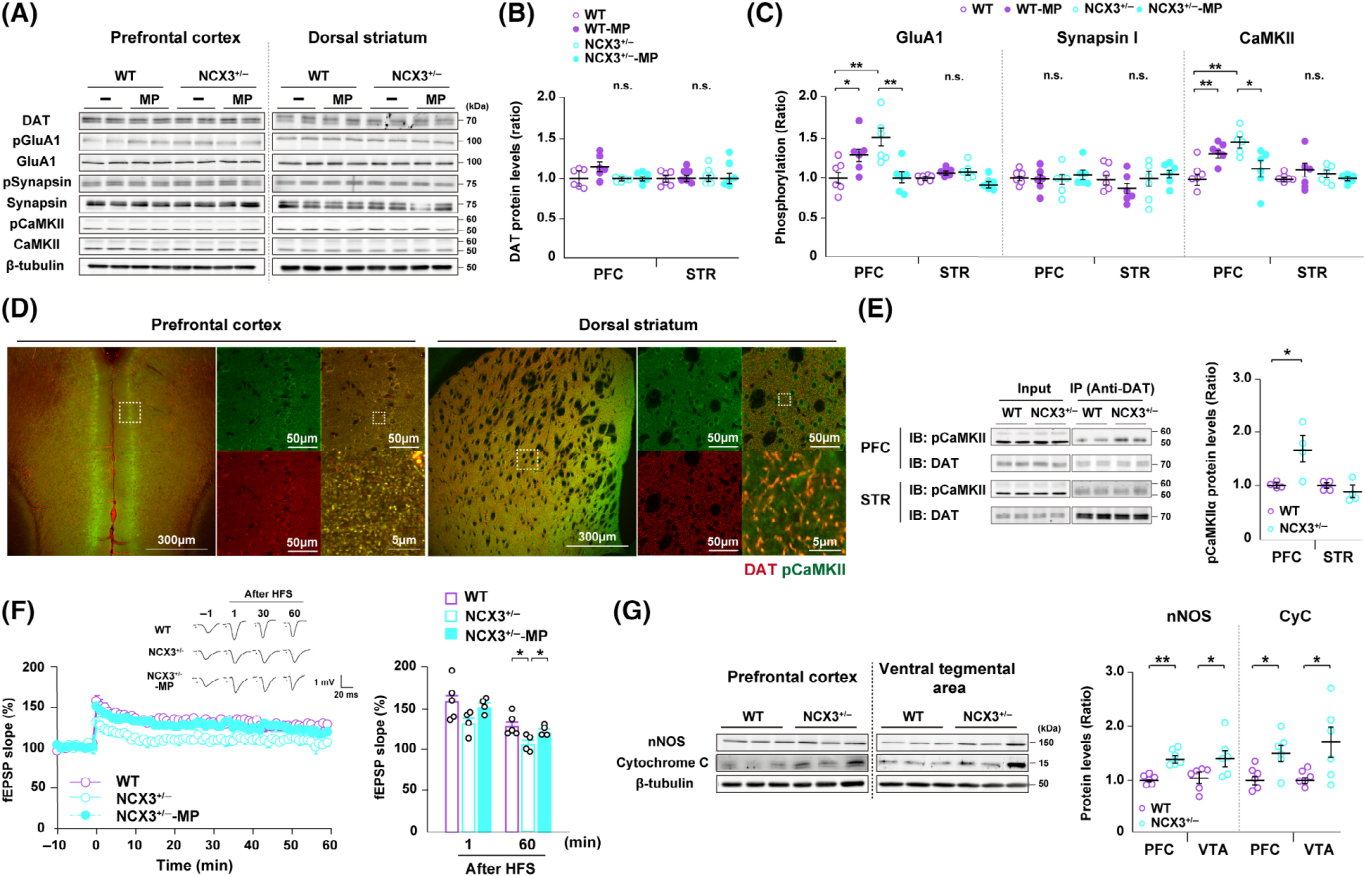
CaMKII excessive activation and strong interaction between DAT as a substrate observed in the PFC of NCX3^+/−^ mice. (A) Representative immunoblots of the PFC and the STR lysates probed with antibodies recognizing DAT, phosphorylated GluA1 (Ser‐831), GluA1, autophosphorylated CaMKIIα (Thr‐286), CaMKII, phosphorylated Synapsin I (Ser‐603), Synapsin I and β‐tubulin. (B) Quantitative analyses of DAT protein levels. (C) Quantitative analysis of protein phosphorylation. CaMKII autophosphorylation and GluA1 phosphorylation, its downstream substrates were significantly increased in the PFC of NCX3^+/−^ mice and methylphenidate treatment significantly rescued excess CaMKIIα (Thr‐286) autophosphorylation and GluA1 phosphorylation in the PFC of NCX3^+/−^ mice (*n* = 6 per group, [CaMKII] *F*_(3,20)_ = 6.78, **P < 0.01 and *P < 0.05, inter‐group comparison, [GluA1] *F*_(3,20)_ = 6.38, **P < 0.01 and *P < 0.05, inter‐group comparison). (D) Confocal microscopy images showing double staining of PFC and STR for autophosphorylated CaMKIIα (Thr‐286) (green) and DAT (red) and merged images. Strong colocalization between phospho‐CaMKIIα and DAT is shown in PFC and STR regions. Scale bars, 300 μm at low magnification and 50 and 5 μm (enlarged) in high magnification images. (E) Representative immunoprecipitation and whole‐cell lysate input for autophosphorylated CaMKIIα (Thr‐286), along with grouped analysis of autophosphorylated CaMKIIα (Thr‐286)–DAT interaction. NCX3^+/−^ mice revealed strong physical interaction between phospho‐CaMKIIα and DAT in the PFC but not in the STR ([PFC] *n* = 4 per group, *t*‐test, *P < 0.05). (F) Representative field excitatory postsynaptic potentials (fEPSPs) recorded from the PFC of WT, NCX3^+/−^, and methylphenidate‐treated NCX3^+/−^ mice. Changes in the slope of fEPSPs following high‐frequency stimulation recorded in the PFC are attenuated in NCX3^+/−^ mice. Treatment of methylphenidate significantly ameliorated impaired LTP in the PFC of NCX3^+/−^ mice ([60 min] *n* = 4–5 each, *F*_(2,10)_ = 5.33, *P < 0.05, inter‐group comparison). Representative trace images from three groups are shown from 4 to 5 independent trials (WT; *n* = 5, NCX3^+/−^; *n* = 5, NCX3^+/−^ MP; *n* = 4). (G) Representative immunoblots of the PFC and the VTA lysates from WT and NCX3^+/−^ mice probed with antibodies recognizing nNOS, cytochrome C, and β‐tubulin. Quantitative analyses from obtained blotting data revealed that nNOS and cytochrome C protein levels were significantly increased in NCX3^+/−^ mice in both the PFC and VTA regions (*n* = 6 per group, *t*‐test, **P < 0.01, *P < 0.05). Data are expressed as the means ± standard error of the mean (SEM). CaMKII, calcium/calmodulin‐dependent protein kinase II; CyC, cytochrome C; DAT, dopamine transporter; fEPSP, field excitatory postsynaptic potentials; HFS, high‐frequency stimulation; MP, methylphenidate; NCX, sodium‐calcium exchanger; nNOS, neuronal nitric oxide synthase; PFC, prefrontal cortex; VTA, ventral tegmental area; WT, wild‐type.

Similar to N27 cells, autophosphorylation of CaMKIIα (Thr‐286) and GluA1 (Ser‐831) phosphorylation was markedly increased in the PFC of NCX3^+/−^ mice relative to that in WT mice ([NCX3^+/−^] CaMKIIα Thr‐286: 1.44 ± 0.07; GluA1 Ser‐831: 1.46 ± 0.12 ratio relative to WT, *n* = 6 each) (Fig. 4A,B). Treatment of methylphenidate significantly suppressed autophosphorylation of CaMKIIα (Thr‐286) and GluA1 (Ser‐831) phosphorylation in the PFC of NCX3^+/−^ mice ([NCX3^+/−^ plus methylphenidate] CaMKIIα Thr‐286: 1.10 ± 0.10; GluA1 Ser‐831: 0.98 ± 0.07 ratio relative to WT, *n* = 6 each) (Fig. 4A,B). In contrast, no significant differences were observed in DAT expression or Synapsin I (Ser‐603) phosphorylation in the PFC among all groups (Fig. 4A,B). Notably, no significant differences were observed in DAT expression or autophosphorylation of CaMKII, GluA1, and Synapsin I phosphorylation among all groups in the STR (Fig. 4A–C).

We further assessed protein–protein interaction between phospho‐CaMKII and DAT in the PFC and STR of WT and NCX3^+/−^ mice. As a premise, we detected the physical interaction between phospho‐CaMKIIα and DAT in the PFC and STR by using immunohistochemistry (Fig. 4D). Subsequently, we found that phospho‐CaMKII in the PFC of NCX3^+/−^ mice interacted more strongly with DAT compared to WT mice, while no differences were observed in the STR due to gene deletion ([PFC] NCX3^+/−^; CaMKIIα Thr‐286: 1.68 ± 0.24 ratio relative to WT, *n* = 4 each) (Fig. 4E).

Neural plasticity in the PFC is considered to be mediated by dopaminergic functions involved in dopamine D1 receptor signaling, including protein kinase A (PKA) [23]. We thereon tested the long‐term potentiation (LTP) in the PFC of WT and NCX3^+/−^ mice (Fig. 4F). In the cortical slices of WT mice, high‐frequency stimulation (100 Hz, two trains) of layers II/III of the prelimbic cortex induced LTP in layer V region, which lasted over 60 min ([WT]: 127.48 ± 3.46% of the baseline at 60 min, *n* = 5) (Fig. 4F). The LTP in the PFC was significantly reduced in NCX3^+/−^ mice relative to WT mice, while reduced LTP was significantly restored by methylphenidate ([NCX3^+/−^]: 106.01 ± 4.91%, [NCX3^+/−^ plus methylphenidate]: 119.00 ± 4.56% of the baseline at 60 min, *n* = 4 each) (Fig. 4F). Since mitochondrial oxidative, metabolic, and calcium buffering functions are candidates for regulating the neural plasticity and transmission, we assessed neuronal nitric oxide synthase (nNOS) and cytochrome C protein expression in the PFC and VTA of WT and NCX3^+/−^ mice. Quantitative analyses obtained from blotting data revealed that nNOS and cytochrome C protein levels were significantly increased in NCX3^+/−^ mice in both the PFC and VTA regions ([nNOS] PFC; NCX3^+/−^, 1.37 ± 0.06; VTA; NCX3^+/−^, 1.39 ± 0.14 [cytochrome C] PFC; NCX3^+/−^, 1.47 ± 0.14; PFC; NCX3^+/−^, 1.69 ± 0.28, *n* = 6 per group) (Fig. 4G).

### NCX3 heterozygous mice exhibit persistent elevation of basal dopamine levels in the PFC vis the dopamine D1 receptor signaling pathway

**Fig. 5:**
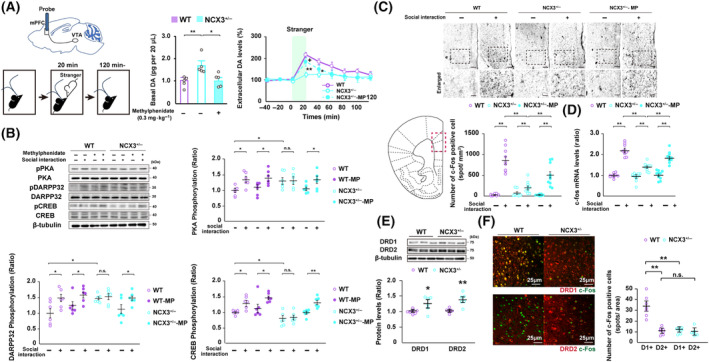
NCX3^+/−^ mice exhibited persistent elevation of basal dopamine levels in the PFC, resulting in activation of dopamine D1 receptor/PKA/DARPP32 signaling pathway. (A) *In vivo* microdialysis task. Left: basal extracellular dopamine levels in the mPFC. Right: Time course of extracellular dopamine levels in the mPFC. Social cue stimulation by unfamiliar novel mice was applied to the subjects for 0–20 min. Error bars represent the standard error of the mean. The basal level of dopamine in the mPFC of NCX3^+/−^ mice was significantly elevated relative to that in WT mice, and methylphenidate treatment rescued the markedly reduced basal dopamine levels (*n* = 5 per group, *F*_(2,12)_ = 7.80, **P < 0.01, and *P < 0.05, inter‐group comparison). The extracellular dopamine levels evoked by social cues stimulation in the mPFC of NCX3^+/−^ mice were significantly decreased relative to WT mice and methylphenidate treatment restored the elevation of extracellular dopamine levels evoked by social cues stimulation (*n* = 5 per group, 20 min after social cues, *F*_(2,12)_ = 9.67, *t*‐test, *P < 0.05 vs WT and ^+^ *P* < 0.05 vs NCX3^+/−^, 40 min after social cues, *F*_(2,12)_ = 3.31, *t*‐test, **P < 0.01 vs WT). (B) Left, representative immunoblots of the PFC lysates probed with antibodies recognizing phosphorylated PKA (Thr‐197), PKA, phosphorylated DARPP‐32 (Thr‐34), DARPP‐32, phosphorylated CREB (Ser‐133), CREB, and β‐tubulin. Right: Quantitative analysis of phosphorylation. Phosphorylation of PKA and DARPP‐32, its downstream substrates were significantly increased in the PFC of NCX3^+/−^ mice. One hour after social stimulation, WT mice showed significantly elevated PKA‐DARPP32‐CREB phosphorylation in the PFC, but not in the PFC of NCX3^+/−^ mice, relative to unstimulated conditions. Treatment of methylphenidate leads to restore the rise in PKA‐DARPP32‐CREB phosphorylation evoked by the social cues stimulation in the PFC of NCX3^+/−^ mice (*n* = 6 per group, [PKA] *F*_(7,40)_ = 2.70, *P < 0.05, inter‐group comparison, [DARPP32] *F*_(7,40)_ = 3.53, *P < 0.05, inter‐group comparison, [CREB] *F*_(7,40)_ = 9.43, **P < 0.01 and *P < 0.05, inter‐group comparison). (C, D) Expression of c‐Fos in the PFC following social cue stimulation by IHC (C) and qRT‐PCR (D). Stimulated WT mice showed a significant increase in c‐Fos expression in the PFC compared to unstimulated control, whereas NCX3^+/−^ mice also showed a significant increase in c‐Fos expression evoked by social cues stimulation but those were not as much as WT mice. Treatment with methylphenidate significantly restored the rise in c‐Fos expression evoked by social cue stimulation. Scale bars, 100 μm at low magnification and 50 μm in enlarged images ([qRT‐PCR] *F*_(5,54)_ = 57.01, **P < 0.01, inter‐group comparison, *n* = 10 each; [IHC] *F*_(5,54)_ = 40.76, **P < 0.01, inter‐group comparison, *n* = 10 each). (E) Representative immunoblots of the PFC lysates from WT and NCX3^+/−^ mice probed with antibodies recognizing DRD1, DRD2, and β‐tubulin. Quantitative analyses from obtained blotting data revealed that both DRD1 and DRD2 protein levels were significantly increased in NCX3^+/−^ mice (*n* = 6 per group, *t*‐test, **P < 0.01, *P < 0.05). (F) Detection of c‐Fos in the dopamine D1‐ or D2‐positive neurons in the PFC region following social cue stimulation by IHC. Confocal microscopy images showing double staining of PFC from WT and NCX3^+/−^ mice for c‐Fos (green) and DRD1 or DRD2 (red) merged images. Scale bars: 25 μm in the images. Stimulated WT mice showed a significantly increased c‐Fos expression in the dopamine D1‐positive neurons (D1+) compared to in D2‐positive neurons (D2+), whereas NCX3^+/−^ mice showed a significant decrease in c‐Fos expression in the dopamine D1‐positive neurons evoked by social cues stimulation compared with those of WT mice (*F*_(3,24)_ = 29.41, **P < 0.01, inter‐group comparison, *n* = 7 each). Data are expressed as the means ± standard error of the mean (SEM). DA, dopamine; DRD1, dopamine receptor D1; DRD2, dopamine receptor D2; MP, methylphenidate; mPFC, medial prefrontal cortex; NCX, sodium‐calcium exchanger; WT, wild‐type.

Next, we characterized the both basal extracellular dopamine levels and social cues‐evoked dopamine release in the PFC of NCX3^+/−^ mice relative to WT mice by an *in vivo* microdialysis analysis. The basal levels of dopamine was significantly elevated in the PFC of NCX3^+/−^ mice relative to WT mice, while treatment of methylphenidate markedly reduced the elevated basal dopamine levels in NCX3^+/−^ mice ([WT]: 0.97 ± 0.11 pg, [NCX3^+/−^]: 1.69 ± 0.19 pg, [NCX3^+/−^ plus methylphenidate]: 1.03 ± 0.13 pg per 20 μL of dialysate, *n* = 5 each) (Fig. 5A). Social cue stimulation by unfamiliar novel mice induced an increase in extracellular dopamine levels in the PFC of WT mice and it significantly suppressed the extracellular dopamine levels responses to the social cue stimulation in the PFC of NCX3^+/−^ mice, conversely (20 min after social cues, [WT]: 215.57 ± 10.07%, [NCX3^+/−^]: 124.18 ± 12.32%, 40 min after social cues, [WT]: 183.49 ± 13.92%, [NCX3^+/−^]: 125.72 ± 13.98%, *n* = 5 each) (Fig. 5A). In addition, treatment with methylphenidate restored the extracellular dopamine levels in response to social cue stimulation to the same levels as in WT mice (20 min after social cues, [NCX3^+/−^ plus methylphenidate]: 183.02 ± 20.30%, *n* = 5 each).

The dopamine D1 receptor signaling pathway is involved in neural plasticity and social behavior [23, 24]. Hence, we evaluated the downstream targets of the dopamine D1 receptor by PKA, DARPP32, and CREB in the PFC extracts prepared with or without social cue stimulation in NCX3^+/−^ mice relative to WT mice. After 1 h after social cues stimulation, phosphorylation of PKA (Thr‐197), DARPP32 (Thr‐34), and CREB (Ser‐133) significantly elevated in the PFC of WT mice ([PKA] WT‐unstimulated; 1.00 ± 0.06, WT‐stimulated; 1.32 ± 0.09, [DARPP32] WT‐unstimulated; 1.01 ± 0.15, WT‐stimulated; 1.47 ± 0.12, [CREB] WT‐unstimulated; 1.00 ± 0.03, WT‐stimulated; 1.29 ± 0.09 ratio relative to WT, *n* = 6 each) (Fig. 5B). In contrast, phosphorylation of PKA (Thr‐197), DARPP32 (Thr‐34), and CREB (Ser‐133) in the PFC failed to elevate with or without social cue stimulation in NCX3^+/−^ mice ([PKA] NCX3^+/−^‐unstimulated; 1.28 ± 0.10, NCX3^+/−^‐stimulated; 1.29 ± 0.10, [DARPP32] NCX3^+/−^‐unstimulated; 1.46 ± 0.05, NCX3^+/−^‐stimulated; 1.52 ± 0.10, [CREB] NCX3^+/−^‐unstimulated; 0.78 ± 0.08, NCX3^+/−^‐stimulated 0.84 ± 0.07 ratio relative to WT, *n* = 6 each) (Fig. 5B). Meanwhile, treatment with methylphenidate failed to further increase the phosphorylation of PKA (Thr‐197), DARPP32 (Thr‐34), and CREB (Ser‐133) evoked by the social cue stimulation in the PFC of NCX3^+/−^ mice ([PKA] NCX3^+/−^ plus methylphenidate‐unstimulated; 1.03 ± 0.06, NCX3^+/−^ plus methylphenidate‐stimulated; 1.32 ± 0.11, [DARPP32] NCX3^+/−^ plus methylphenidate‐unstimulated; 1.11 ± 0.14, NCX3^+/−^ plus methylphenidate‐stimulated; 1.47 ± 0.08, [CREB] NCX3^+/−^ plus methylphenidate‐unstimulated; 0.99 ± 0.05, NCX3^+/−^ plus methylphenidate‐stimulated; 1.31 ± 0.07, *n* = 6 each) (Fig. 5B).

c‐Fos is an immediate early response gene that is considered a marker of neural excitation and transcription via CREB phosphorylation (Ser‐133) [25]. We next determined the protein and mRNA expression of c‐Fos in the PFC following social cue stimulation by both quantitative reverse transcription polymerase chain reaction (qRT‐PCR) and immunohistochemistry (IHC). Similar to PKA (Thr‐197), DARPP32 (Thr‐34) and CREB (Ser‐133) phosphorylation was elevated following social cue stimulation in the PFC of WT mice; the PFC of WT mice exhibited remarkable increase in c‐Fos expression followed by social cue stimulation. In addition, the PFC of NCX3^+/−^ mice also exhibited significantly increased c‐Fos expression following social cue stimulation. However, the expression of c‐Fos in PFC of NCX3^+/−^ mice following social cue stimulation were not increased nearly as much as those of the WT mice ([qRT‐PCR] WT‐unstimulated; 1.00 ± 0.03, WT‐stimulated; 2.18 ± 0.09, NCX3^+/−^‐unstimulated; 0.94 ± 0.06, NCX3^+/−^‐stimulated; 1.39 ± 0.06, ratio relative to WT, *n* = 10 each, [IHC] WT‐unstimulated; 20.00 ± 5.17, WT‐stimulated; 849.40 ± 86.18, NCX3^+/−^‐unstimulated; 47.70 ± 24.86, NCX3^+/−^‐stimulated; 201.70 ± 46.49 as the number of positive cells, *n* = 10 each) (Fig. 5C,D). The moderate induction of c‐Fos expression after social cue stimulation in the PFC of NCX3^+/−^ mice was restored upon methylphenidate treatment ([qRT‐PCR] NCX3^+/−^ plus methylphenidate‐unstimulated; 1.01 ± 0.07, NCX3^+/−^ plus methylphenidate‐stimulated; 1.84 ± 0.09 ratio relative to WT, *n* = 10 each, [IHC] NCX3^+/−^ plus methylphenidate‐unstimulated; 17.80 ± 5.87, NCX3^+/−^ plus methylphenidate‐stimulated; 499.70 ± 80.45 as the number of positive cells, *n* = 10 each) (Fig. 5C,D).

Because a sustained increase in prefrontal dopamine levels reportedly suppresses c‐Fos induction triggered by social stimuli in neurons expressing dopamine D1 receptors, but not D2 receptors, we subsequently confirmed the characteristics of dopamine D1 and D2 receptors in the PFC of NCX3^+/−^ mice [24]. The protein expression of both dopamine D1 and D2 receptors were significantly increased in the PFC of NCX3^+/−^ mice (DRD1; NCX3^+/−^, 1.26 ± 0.10; DRD2; NCX3^+/−^, 1.39 ± 0.08, *n* = 6 each) (Fig. 5E). Meanwhile, the number of c‐Fos‐positive cells colocalized with dopamine D1 but not D2 receptors in the PFC was significantly increased in NCX3^+/−^ mice following social cue stimulation ([WT] D1+; 33.57 ± 3.71, D2+; 10.57 ± 1.09 [NCX3^+/−^] D1+; 11.57 ± 1.04, D2+; 9.57 ± 1.21, *n* = 7 each) (Fig. 5F).

## Discussion

The pathology of ADHD is believed to be a result of abnormalities in catecholamine transmission to the PFC, which corresponds to the key functions of motivation, performance, and learning, and is most developed in human beings compared to animals. Here, we documented that NCX3 is abundantly localized in dopaminergic neurons distributed within the VTA, the region that projects dopaminergic inputs into the cortical limbic area, including the PFC (Fig. 1D). Furthermore, NCX3 mRNA levels were significantly higher levels in VTA and N27 dopaminergic neuron compared with other NCX isoforms (Figs 1C and 2A). In contrast, NCX3 has sparse expression in the SNc, the region that projects dopaminergic input to the striatal area (Fig. 1A,B). Previous studies have reported that the chemogenetic activation of dopamine neurons in the VTA induces pronounced and long‐lasting hyperactive behavior in rodents, whereas chemogenetic activation of dopamine neurons in the SNc induces a modestly increased in locomotion [26]. Continuous chemogenetic activation of VTA dopaminergic neurons has been reported to increase resting‐state dopamine levels in the medial prefrontal cortex [24]. Hence, prolonged hyperlocomotion observed in NCX3^+/−^ mice may be due to the excess extracellular dopamine levels sent to the PFC due to the tonic excitation of dopaminergic neuron projected from VTA (Figs 1A,B, 3A, and 5A).

Physiologically, dopamine D1 receptor‐expressing neurons have a lower hyperpolarization‐activated nonspecific cation current and little rebound depolarization relative to dopamine D2 receptor‐expressing neurons in the PFC. These neuronal properties are modulated by dopamine through the differential activation of dopamine D1 and D2 receptors [27, 28, 29]. Furthermore, social memory‐associated neurons in the medial PFC (mPFC) were also reported to display the electrophysiological properties of the dopamine D1 receptor‐expressed neuron [30, 31]. In addition, Xing *et al*. [32] demonstrated enhanced AMPAR‐mediated synaptic transmission in layer 5 PL pyramidal neurons of socially dominant mice, and this phenomenon mainly existed in the dopamine D1 receptor but not in the dopamine D2 receptor‐expressing neurons in the mPFC. Consistent with these physiological changes, Sotoyama *et al*. [24] reported that a sustained increase in prefrontal dopamine levels suppressed c‐Fos induction triggered by social stimuli in neurons expressing dopamine D1 receptors, but not D2 receptors. Similar to previous study, we determined c‐Fos induction triggered by social stimuli occurred primarily in neurons expressing dopamine D1 receptors, but not D2 receptors in WT mice (Fig. 5F). Conversely, c‐Fos induction in dopamine D1 receptor‐positive neurons by social stimuli was significantly decreased in NCX3^+/−^ mice (Fig. 5F). Therefore, the one possible mechanism of social dysfunction in NCX3^+/−^ mice has been proposed to be the persistent activation of postsynaptic dopamine D1 receptor according to the sustained elevated dopamine levels. However, the sustained elevated dopamine levels and social stimuli‐induced c‐Fos induction observed in the PFC of NCX3^+/−^ mice were restored by methylphenidate treatment (Fig. 5A,C). Hence, appropriate dopamine levels in the PFC would be essential for the regulation of dopamine D1 receptor‐positive neuronal activity, which in turn leads to the maintenance of appropriate social interactions.

Cognitive function is essential for dopaminergic neurotransmission in the PFC For instance, increased dopamine turnover or supranormal stimulation of dopamine D1 receptors in the PFC impairs spatial working memory performance in rodents [33, 34]. Indeed, impaired working memory with markedly decreased LTP in the PFC along with significantly increased DARPP‐32 phosphorylation (Thr‐34) in the striatum have been observed in DAT‐KO mice [35, 36, 37]. As we report here, NCX3^+/−^ mice exhibited the cognitive dysfunction through the sustained activation of dopamine D1 receptor signaling pathway in the PFC, whereas the cognitive dysfunction observed in NCX3^+/−^ mice were ameliorated by administration of methylphenidate via DAT‐regulated alternation of dopamine turnover (Figs 3E–G and 5B). Consistent with dopamine D1 receptor signaling pathway in the PFC, reduced prefrontal LTP in NCX3^+/−^ mice become significantly restored by methylphenidate (Fig. 4F). Thus, a normalization of the dopamine D1 receptor signaling pathway in the PFC may play an essential role in restoring injured LTP and cognitive dysfunction.

DAT is implicated in ADHD pathophysiology not only because gene variants serve as ADHD risk alleles, but also because blockage of DAT serves as a therapeutic target for ADHD. Recent work has shown that hDAT A559V, a rare coding variant of ADHD, elicits an anomalous DAT‐mediated dopamine efflux, similar to that caused by amphetamine‐like psychostimulants [18]. In addition, the anomalous dopamine efflux via DAT in response to amphetamine is induced by stimulation of CaMKIIα through binding the C terminus of DAT in dopaminergic neurons [21, 38]. In Fig. 2I,J, CaMKIIα autophosphorylation strongly bound to the C terminus of DAT and decreased dopamine intake in siNCX3 N27 cells. Furthermore, we observed that inhibition of dopamine intake in siNCX3 N27 cells was rescued by pretreatment with KN93, an inhibitor of CaMKII (Fig. 2K). Thus, one possible mechanism for decreased dopamine intake in siNCX3 N27 cells could be the excessive CaMKII autophosphorylation‐induced dopamine efflux response (Fig. 2K).

Here, we also observed increased CaMKIIα autophosphorylation in the siNCX3 N27 cells and the PFC lysates from NCX3^+/−^ mice (Figs 2F,G and 4A,B). CaMKII is preferentially activated by cytosolic Ca^2+^ concentrations (*K*_d_ = approximately 40–100 nm) and the range of intracellular Ca^2+^ concentrations oscillate from 10 to 100 nm under resting conditions [39, 40]. Under NCX3 knockdown conditions in cultured cerebellar granule neurons, glutamate‐induced increases in intracellular Ca^2+^ irreversibly remained well above normal recovery levels [41]. Likewise, NCX3 knockdown skeletal muscle fibers induced a slight but significant increase in both the intracellular Ca^2+^ amplitude and the areas of the curve following high potassium depolarization [42]. Similar to previous studies, we detected a significant increase in basal [Ca^2+^]_i_ in siNCX3 N27 cells compared to control N27 cells (Fig. 2E). Thus, persistent Ca^2+^ elevation following neuronal depolarization may cause aberrant elevation of CaMKIIα autophosphorylation in the PFC of NCX3^+/−^ mice (Fig. 4A,B). Multiple studies have shown that elevated CaMKIIα autophosphorylation is associated with disruption of cognition and synaptic plasticity. A knock‐in mouse in which the autophosphorylation site at both T286A and T305D of CaMKIIα shows impairment in hippocampal LTP and learning, respectively [43, 44]. In addition, α‐thalassemia X‐linked mental retardation mutant mice show cognitive impairment with markedly increased CaMKIIα autophosphorylation (Thr‐286) levels in the PFC and Ube3a maternal‐deficient mice also show similar result in impaired memory learning and LTP with markedly increased CaMKIIα autophosphorylation (Thr‐305) levels in the hippocampus [45, 46, 47, 48]. Therefore, NCX3 likely plays a role in synaptic plasticity and cognitive function regulated by CaMKIIα autophosphorylation through varying intracellular Ca^2+^ concentration (Fig. 4A,B,F).

Our study demonstrated that DAT inhibition by methylphenidate rescued hyperactivity, inattention and cognitive deficit which then suppressed basal dopamine levels in the PFC of NCX3^+/−^ mice (Figs 3A,D–G and 5A). In addition, we also elucidated that acute exposure to methylphenidate at 3.0 mg·kg^−1^ i.p. normalized CaMKIIα autophosphorylation in the PFC of NCX3^+/−^ mice (Fig. 4A,B). In sync with our results, acute exposure to methylphenidate at 1 mg·kg^−1^ p.o. suppressed CaMKIIα autophosphorylation in the medial PFC of spontaneously hypertensive rats, which widely used as an animal model of ADHD [49]. Different conformational states of DAT are stabilized by typical (cocaine‐like) DAT blockers, including methylphenidate, which preferred its interaction with CaMKII [19]. Thus, it was hypothesized that aberrant CaMKII downstream activation of mobilization of the reserve pool of DA vesicles results in a decline in DA release from presynaptic terminals; however, this effect can be blocked by methylphenidate through the inhibition of CaMKII (Figs 4A,C,E and 5A) [50]. Unpredictably, we determined that Synapsin I (Ser‐603), a substrate of CaMKII, has no significant effect among the vehicle‐ or methylphenidate‐treated in WT and NCX3^+/−^ mice groups. Accordingly, CaMKII downstream activation of Synapsin I‐dependent clustering of DA vesicles did not account for the modulation of DA release by methylphenidate or NCX3 knockdown (Fig. 4A,C).

Presynaptic dopamine D2 receptors can also change dopamine transmission through the inhibition of tyrosine hydroxylase. Reduction of tyrosine hydroxylase activity likely occur via an inhibition of AC‐cAMP‐PKA signaling‐mediated regulatory domain of tyrosine hydroxylase. Downregulation of tyrosine hydroxylase following prolonged presynaptic dopamine D2 receptor activation by methylphenidate may lead to reductions in the filling dopamine vesicles and hyperactivity [51, 52]. In addition, methylphenidate also reported to improve prefrontal cortical cognitive function through stimulation of α2 adrenoceptor in rodent [53]. Thus, another possible mechanism of action of methylphenidate in terms of amelioration of hyperactivity and cognitive dysfunction could be due to altered dopamine D2 and α2 adrenergic receptor properties.

The augmentation of dopamine release induced by psychostimulants enhances the heteroreceptor complex formation between postsynaptic dopamine D2 receptors and NMDA receptor NR2B subunits, which likely leads to reduced NR2B phosphorylation at a CaMKII‐sensitive site (Ser‐1303) due to the disruption of CaMKII‐NR2B and inhibition of NMDA receptor‐mediated currents [54]. Activation of dopamine D2 receptors account for decrease in PKA phosphorylation which lead to suppression of DARPP‐32 (Thr‐34)/PP1α signaling, whereas dopamine D1 receptors act in an opposite manner to regulate PKA/DARPP‐32/PP‐1 signaling [55, 56]. Besides, NCX3 is also expressed and functionally active on the outer mitochondrial membrane where it works in concert with the PKA anchoring protein AKAP121 in promoting mitochondrial calcium efflux and metabolic activity [57] Based on the above research, NCX3^+/−^ mice showed a significant increase in nNOS and cytochrome C protein levels in mPFC and VTA compared to WT mice, resulting in mitochondrial dysfunction manifested by the decrease of redox and Δψm which will inhibit the energy providing to support neurotransmitter release at synaptic level (Fig. 4G). In addition, we detected a significant increase in the protein expression of both dopamine D1 and D2 receptor in mPFC of NCX3^+/−^ mice, although it should be noted that our method cannot distinguish between receptor expression patterns in pre‐ and postsynaptic regions (Fig. 5E). However, an imbalance between postsynaptic dopamine D1 and D2 receptors pathway may contribute critically to abnormal activation of CaMKII and PKA‐DARPP‐32 signaling in the mPFC of NCX3^+/−^ mice (Fig. 5B).

**Fig. 6:**
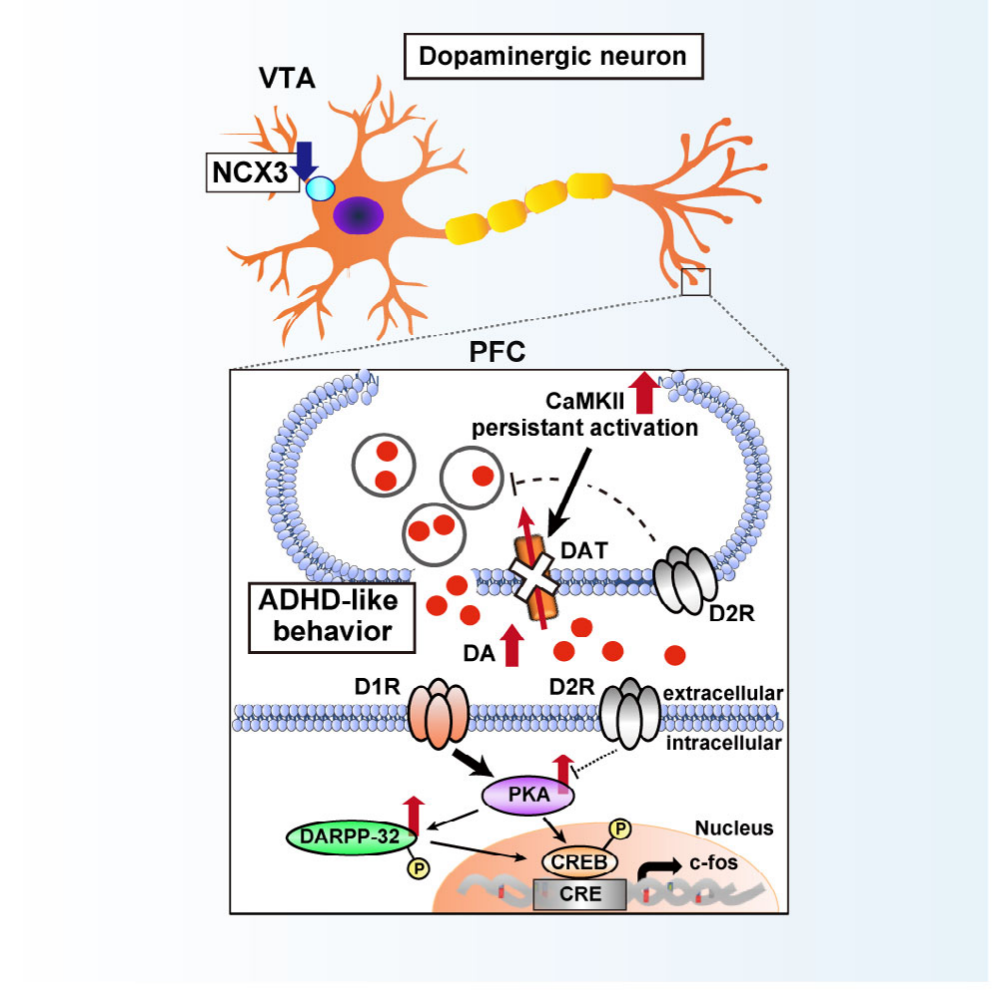
Proposed mechanism of ADHD‐like behavior presented in NCX3^+/−^ mice. Results suggest that NCX3 knockdown in dopaminergic neurons of the VTA induces a direct physical interaction between phospho‐CaMKII and DAT, inducing excess extracellular dopamine levels due to the disruption of dopamine clearance in the PFC. In concordance with the increase of extracellular dopamine levels in the PFC, NCX3^+/−^ mice exhibited the activation of dopamine D1 receptor signaling pathways, which promote hyperactivity, cognitive deficit, social dysfunction associated with ADHD. ADHD, attention‐deficit/hyperactivity disorder; CaMKII, calcium/calmodulin‐dependent protein kinase II; D1R, dopamine D1 receptor; D2R, dopamine D2 receptor; DA, dopamine; NXC, sodium‐calcium exchanger; PFC, prefrontal cortex; VTA, ventral tegmental area.

In conclusion, we demonstrated that NCX3^+/−^ mice exhibited ADHD‐like symptoms underlying prefrontal dopaminergic dysfunction. We also concluded that NCX3 knockdown‐induced excessive CaMKII activation and a concomitantly strong interaction with DAT as a substrate, in turn evokes aberrant spatial and temporal dynamics of dopamine neurotransmission in dopaminergic neurons. In addition, behavioral abnormalities as well as underlying molecular properties in the PFC of NCX3^+/−^ mice were ameliorated by methylphenidate. Under these circumstances, pursuing the mechanisms of NCX3‐dependent prefrontal dopaminergic dysfunction will provide new insights to realize the neurobiological mechanistic molecular pattern underlying neuropsychiatric disorders, including ADHD (Fig. 6).

## Materials and methods

### Animals

NCX1, NCX2, and NCX3 heterozygous mice (NCX1–3^+/−^: aged 10 weeks old; male mice: NCX1^+/−^ *n* = 6, NCX2^+/−^ *n* = 6, NCX3^+/−^ *n* = 30) and age‐matched WT male mice (C57BL/6J, *n* = 30) were used in the experiments. C57BL/6 mice were purchased from Japan‐SLC (Shizuoka, Japan), and the NCX1^+/−^, NCX2^+/−^, and NCX3^+/−^ mice were generated as reported previously [58, 59, 60]. During the procedures, all animals had access to food and water *ad libitum* at controlled temperature (23 ± 1 °C) and humidity (55 ± 5%), under a 12 h light/dark cycle (lights on at 9 a.m.). All animal experimental procedures were approved by the Committee on Animal Experiments at Tohoku University, Japan, according to the NIH guidelines (no. 2019PhA‐003‐02).

### Drug treatment

Mice were intraperitoneally treated with methylphenidate or vehicle (saline) 12 h before initiating the experiments.

### Behavioral tests

#### Spontaneous locomotor activity

The detailed protocol has been previously described [61]. To measure spontaneous locomotor activity, mice were housed in individual cages and placed below an automated infrared beam‐based monitors (Digital Acquisition System; Neuroscience, Inc., Tokyo, Japan). During the experimental period, all mice were singly housed with *ad libitum* access to food/water under a 12 h light/dark cycle (lights on at 9 a.m.). Locomotor activity data were collected every 1 h throughout the 24 h period.

#### Y‐maze task

Spontaneous alternation behavior in the Y‐maze task reveals short‐term spatial reference memory. The protocol has been detailed previously [62]. The apparatus consisted of three identical arms (50 × 16 × 32 cm) made of black Plexiglas. During the experimental period, each mouse was placed at the end of one arm and allowed to move freely through the maze for an 8‐min session. The percentage of spontaneous alternation behavior was calculated as follows: spontaneous alternation behavior (%) = (actual alternations/total number of arms entered minus two) × 100.

#### Novel object recognition task

The novel object recognition task is based on the tendency of rodents to spend more time exploring a novel object. The protocol has been detailed previously [62]. Mice were individually habituated to an open‐field box (35 × 25 × 35 cm) for two consecutive days. The acquisition trial involved visual exploration of two identical objects for 5 min. After 1 h, the test trial involved replacing one object with a novel object, which was allowed to explore freely for 5 min. The number of approaches taken toward the two objects was scored.

#### Step‐through passive avoidance task

The detailed protocol has been previously reported [62]. This task is based on the innate aversion to the rodento‐illuminated area. The apparatus consisted of a two‐compartment chamber consisting of dark (25 × 25 × 25 cm) and light (14 × 10 × 25 cm) boxes with a floor of stainless‐steel rods. The rods in the dark box were connected to an electronic stimulator (Nihon Kohden Co., Ltd., Tokyo, Japan). The mice were habituated to the apparatus the day before the training trials. During the training trials, a mouse was placed in the light box; when it entered the dark compartment, the door was closed to prevent escape, and the animal received an electric shock (0.4 mA for 500 ms) from the floor for 30 s. After 24 h of training, the latency to enter the dark box was measured as an index of memory retention.

#### Reciprocal social interaction task

To measure reciprocal social interaction behavior, same‐sex/same‐genotype pairs consisting of non‐littermate mice were allowed to interact socially for 5 min after one mouse in the pair was habituated to the test environment for 1 min. The duration and number of social behaviors, including close following, touching, nose‐to‐nose sniffing, nose‐to‐anus sniffing, grooming, and crawling over or under each other, were measured. Prior to testing, mice were socially isolated for 24 h to enhance their level of social motivation. Same‐treatment/same‐genotype pairs consisting of non‐litter mates were used.

#### Rotarod task

The rotarod task was used to assess motor coordination and balance in rodents. Mice were placed in a drum (MK‐630B; Muromachi Kikai. Co., Ltd., Tokyo, Japan) rotating at 20 r.p.m., and the latency to fall was recorded with a maximum cutoff of 5 min.

#### Beam‐walk task

The apparatus consisted of a 1 cm square stainless beam 105 cm in length. The beam was suspended at 49 cm above the floor of the test chamber. The mice were habituated to the goal chamber for 3 min and then placed 10 cm away from the goal box. Once the beam traversed the goal box at a distance of 10 cm, the mice were placed 30, 50, and 80 cm from the goal box and trained to traverse the beam. During the test trials, the number of foot slips while crossing the beam was recorded.

### Electrophysiology

The detailed protocol has been previously described [63]. Briefly, acute PFC slices were prepared by sectioning tissue at a thickness of 400 μm by using vibratome (VT1000S; Leica Microsystems, Wetzlar, Germany). The coronal slices including the PFC region were incubated in oxygenated (95% O_2_ and 5% CO_2_) artificial cerebrospinal fluid at room temperature for a minimum of 2 h. Slices were transferred to an interface recording chamber and perfused at a flow rate of 2 mL·min^−1^ with warmed artificial cerebrospinal fluid (34 °C). Field excitatory postsynaptic potentials were recorded from the layer V region of the prelimbic cortex using a glass electrode filled with 3 m NaCl, and the stimuli were applied at a frequency of 0.05 Hz by using bipolar stimulating electrode that was placed in layer II/III. High‐frequency stimulation of 100 Hz with a 1 s duration was applied twice with a 10 s interval to evoke the long‐term potentiation.

### Cell culture and transfection

N27 rat dopaminergic neural cell line was purchased from Merck Millipore (SCC048). N27 cells were cultured in RPMI1640 supplemented with 10% ES Cell qualified fetal bovine serum, 1% penicillin/streptomycin, and 1% l‐Glutamate Solution (×100) at 37 °C with 5% CO_2_. Twenty‐four hours post seeding, cultured medium was changed to cultured medium with 60 μg·mL^−1^ DHEA and 2 mg·mL^−1^ dibutyryl cyclic AMP for 72 h to induce the differentiation. Differentiated N27 cells were transfected with mock or siNCX3 (sc‐44911; Santa Cruz Biotechnology, Dallas, TX, USA), and the cells were grown for 24 h after transfection before the experiment.

### Immunoblot analysis

Immunoblot analysis was performed as previously described [64]. The following antibodies were used: anti‐NCX1 (1 : 1000 [65]), anti‐NCX2 (1 : 1000 [65]), anti‐phospho‐CaMKII (Thr‐286; 1 : 5000; 12716; Cell Signaling Technology, Danvers, MA, USA), anti‐CaMKII (1 : 5000; ab52476; Abcam, Cambridge, UK), anti‐phospho‐Synapsin I (Ser‐603; 1 : 2000; Merck Millipore), anti‐Synapsin I (1 : 2000; 20258; Protein Tech Group, Rosemont, IL, USA), anti‐phospho‐GluA1 (Ser‐831; 1 : 1000; 04‐823; Merck Millipore), anti‐GluA1 (1 : 1000; AB1504; Merck Millipore), anti‐phospho‐CREB (Ser‐133; 1 : 1000; 9191; Merck Millipore), anti‐CREB (1 : 1000; 4820; Merck Millipore), anti‐DAT (1 : 1000; PA1‐4656; Invitrogen, Waltham, MA, USA), anti‐phospho‐PKA (Thr‐197; 1 : 1000; 4781; Cell Signaling Technology), anti‐PKA (1 : 1000; 5842; Cell Signaling Technology), anti‐phospho‐DARPP32 (Ser‐660; 1 : 1000; IMG‐5373; Novus Biologicals, Centennial, CO, USA), anti‐DARPP32 (1 : 1000; IMG‐5041; Novus Biologicals), anti‐DRD1 (1 : 1000; sc‐33660; Santa Cruz), anti‐DRD2 (1 : 1000; sc‐5303; Santa Cruz), anti‐nNOS (1 : 1000; 18934; Protein Tech Group), anti‐Cytochrome C (1 : 1000; 66264; Protein Tech Group), and anti‐β‐tubulin (1 : 5000; 66420; Protein Tech Group). The fluorescence intensity of the cell‐bound antibodies was visualized using an enhanced chemiluminescence detection system (Bio‐Rad Laboratories, Hercules, CA, USA) and analyzed semi‐quantitatively using the NIH Image program.

A detailed quantification method for analyzing protein phosphorylation (Ratio) is described below. First, the intensity of each protein band was divided by the mean band intensity value of the groups. The calculated value for each sample was then expressed as the ratio changes in the control group (non‐transfected N27 cell, WT mice). Subsequently, the protein phosphorylation values were determined by calculating the phospho‐protein/total‐protein ratio using the normalized values. Finally, the resulting ratios of each sample were expressed as ratio changes of control group.

Co‐immunoprecipitation was performed using extracts from the PFC and striatum samples and the N27 cells. Briefly, extracts containing 50 μg protein were incubated over night at 4 °C with 10 μL of anti‐DAT antibody (Invitrogen) and the immunoprecipitates were attached to 50% protein A‐Sepharose CL‐4B suspension (Cytiva, Tokyo, Japan). After the centrifugation, the immunoprecipitates were washed four times with buffer C (50 mm Tris/HCl (pH 7.5), 0.5 m NaCl, 4 mm EDTA, 4 mm EGTA, 1 mm Na_3_VO_4_, 50 mm NaF, 1 mm DTT, 1 mm PMSF, 2 μg·mL^−1^ pepstatin A, 1 μg·mL^−1^ leupeptin and 100 nmol·L^−1^ calyculin A), and washed twice with 20 mm Tris/HCl (pH 7.5) plus 1 mm DTT. The prepared samples were subjected to 9% acrylamide SDS/PAGE. Cell extracts and immunoprecipitates were analyzed by immunoblotting as described above. The antibodies used were anti‐phospho‐CaMKII (1 : 500; Cell Signaling Technology) and anti‐DAT antibodies (Invitrogen).

### Measurement of dopamine intake in cultured cell

To assess the ability to intake the dopamine to cell, N27 dopaminergic cells were added DA tracer with cultured medium to a final concentration of 0–100 μm, an alkyne‐tagged dopamine, for 30 min at 37 °C. The culture medium was removed, and the cells were washed with PBS twice and replace the PBS with 4% PFA for 15 min at room temperature. PFA (4%) was then treated with a permeabilization/blocking solution containing 10% normal goat serum and 0.5% Triton X‐100 in PBS for 20 min at room temperature. After washing cells with PBS twice, the cells were treated with Click‐iT reaction cocktail which were consisted with 10 μm of Alexa Fluor azide (Lumiprobe Corporation, Hunt Valley, MD, USA) in Click‐iT cell reaction buffer supplemented with the reducing reagent and 4 mm CuSO_4_ (Vector Laboratories, Burlingame, CA, USA) for 30–60 min at room temperature. After the cells were washed, fluorescence was monitored using a fluorescence multimode microplate reader (Nivo™; PerkinElmer, Inc., Waltham, MA, USA).

### Immunohistochemistry

Fluorescent IHC was performed as previously described [12]. The mice were anesthetized with sevoflurane and perfused with phosphate‐buffered saline (PBS; pH 7.4) via the ascending aorta. The perfuse solution was then switched to phosphate buffer (pH 7.4) containing 4% paraformaldehyde. After the perfusion fixation procedure, the brain was removed and post‐fixed in the same solution for 24 h at 4 °C, and brains were sectioned on a vibratome of 50 μm thickness (Leica VT1000S). Coronal sections were incubated for 30 min in PBS, 30 min in 2 N HCl, 1 h in blocking solution (Blocking One Histo; Nacalai Tesque, Inc., Kyoto, Japan), and then overnight in blocking solution at 4 °C with combinations of the following antibodies: rat anti‐NCX3 (1 : 200 [65]), rabbit anti‐ Tyrosine Hydroxylase (1 : 200; AB152; Merck Millipore), mouse anti‐phospho‐CaMKII (Thr‐286; 1 : 200; sc‐32289; Santa Cruz), rabbit anti‐DAT (1 : 200; PA1‐4656; Invitrogen), anti‐DRD1 (1 : 1000; sc‐33660; Santa Cruz), anti‐DRD2 (1 : 1000; sc‐5303; Santa Cruz), and c‐Fos antibody (1 : 200; 2250; Cell Signaling Technology). After thoroughly washing with PBS, the sections were incubated for 3 h in Alexa 488‐labeled anti‐rat IgG and Alexa 546‐labeled anti‐rabbit IgG antibodies. After several PBS washes, the sections were mounted on slides using VECTASHIELD (Vector Laboratories). Immunofluorescence images were obtained using a confocal laser scanning microscope (Nikon AX; Nikon, Tokyo, Japan).

The DAB immunohistochemistry protocol has been reported previously [64]. Coronal sections were incubated for 30 min in PBS, 30 min in 2 N HCl, 1 h in blocking solution (Blocking One Histo), and then overnight in blocking solution at 4 °C with mouse monoclonal c‐Fos antibody (1 : 200; sc‐166940; Santa Cruz). After this step, the sections were incubated with biotin‐conjugated goat anti‐mouse IgG (H + L) secondary antibody (1 : 500; 115‐65‐003; Jackson ImmunoResearch Inc., West Grove, PA, USA). Immunoreactivity was visualized using a VECTASTAIN ABC kit (Vector Laboratories). Images were captured and analyzed under a microscope (Primovert; Carl Zeiss, Jena, Germany). The number of c‐Fos‐positive cells was determined from the central nucleus of the infralimbic cortex on both sides of the brain and averaged over eight sections per appropriate area (mm^2^).

### Immunocytochemistry

Transfected or untransfected N27 dopaminergic cells were fixed in 4% paraformaldehyde, permeabilized with 0.3% Triton X‐100, blocked in 1% BSA for 60 min, and then incubated overnight at 4 °C combinations of the following antibodies: mouse anti‐phospho‐CaMKII (Thr‐286; 1 : 200; sc‐32289; Santa Cruz), rabbit anti‐DAT (1 : 200; PA1‐4656; Invitrogen), and goat anti‐NCX3 (1 : 200; sc‐48896; Santa Cruz). After thorough washing with PBS, the sections were incubated for 3 h with Alexa 488‐labeled anti‐rabbit IgG and Alexa 568‐labeled anti‐mouse IgG antibodies. After several PBS washes, the sections were mounted on slides using VECTASHIELD (Vector Laboratories). Immunofluorescent images were analyzed using a confocal laser scanning microscope (Nikon).

### Measurement of intracellular Ca^2+^ in cultured cells

The detailed protocol has been previously described [66, 67]. Briefly, to assess the [Ca^2+^]_i_, transfected N27 dopaminergic cells were loaded with Fura‐2 AM, a Ca^2+^‐sensitive dye, for 30 min at 37 °C. After dye loading, the cells were washed, and fluorescence was detected using a fluorescence multimode microplate reader (Nivo™; PerkinElmer, Inc.). Fura‐2 loaded cells were excited alternately at 340 and 380 nm, and fluorescent emission was collected at 510 nm. The ratio of fluorescence emissions from excitation at 340 and 380 nm was used to determine [Ca^2+^]_i_. The [Ca^2+^]_i_ was calculated using the following formula:

$${\left[{\mathrm{Ca}}^{2+}\right]}_{i}=\begin{array}{l} K_{d}\times \left(R-R_{\min}\right)/\left(R_{\max}-R\right)\\ \times \left(F_{\min}\left(380\right)/F_{\max}\left(380\right)\right). \end{array}$$

*R* is the ratio of the fluorescence intensity at 340 nm to the intensity at 380 nm. The maximal fluorescence ratios (*R*_max_) were obtained by adding 2 mm CaCl_2_ plus 5 μm ionomycin, and the minimal fluorescence ratios (*R*_max_) were obtained with the further addition of 20 mm EGTA/Tris. *K*_d_ represents the dissociation constant of Fura‐2; 224 nm was used in the present study. All equipment was controlled using the myassays software (PerkinElmer, Inc.).

### Real‐time RT‐PCR

Real‐time RT‐PCR was performed as previously described [68]. Total PFC, substantia nigra, ventral tegmental area, and N27 dopaminergic cells RNA were extracted using TRI reagent (Sigma‐Aldrich, St. Louis, MO, USA) according to the manufacturer's instructions. Real‐time PCR was performed in 96‐well PCR plates (CFX Connect; Bio‐Rad Laboratories) using KAPA SYBR Fast qPCR (Nippon Genetics Co., Ltd., Tokyo, Japan). Primer sequences were as follows: mouse NCX1, 5′‐CTTCGTCCCACCTACAGAAT‐3′ and 5′‐TGGTAGATGGCAGCAATGGA‐3′; mouse NCX2, 5′‐TGCCATCCTGCTTTGACTAC‐3′ and 5′‐GTGAACAGTGTGACCGAGAA‐3′; mouse NCX3, 5′‐GAAACATGCAGCAGAGCAAG‐3′ and 5′‐GACATTGCTCAGTCTCACGA‐3; mouse c‐fos, 5′‐CGAAGGGAACGGAATAAGATG‐3′ and 5′‐GCTGCCAAAATAAACTCCAG‐3; and mouse glyceraldehyde 3‐phosphate dehydrogenase, 5′‐TGTGTCCGTCGTGGATCTGA‐3′ and 5′‐CACCACCTTCTTGATGTCATCATAC‐3′.

### *In vivo* microdialysis

*In vivo* microdialysis was performed as detailed previously [69]. The guide cannula was placed in the mPFC (AP, +1.6 mm, ML, +0.3 mm, DV, −1.6 mm). The probe was perfused with Ringer's solution at a flow rate of 1.0 μL·min^−1^, and collected fraction was injected into the high‐performance liquid chromatography system for 20 min intervals (HTEC‐500; Eicom, Kyoto, Japan). After 1 h of equilibration, seven dialysates were collected every 20 min, followed by social cues from the novel stranger mice.

### Data analysis

Data are expressed as the means ± standard error of the mean (SEM). Statistical analyses were performed using prism 6 software (GraphPad Software, San Diego, CA, USA). Comparisons between the two experimental groups were performed using an unpaired *t*‐test. The statistical significance of differences among the groups was tested using one‐way or two‐way analysis of variance, followed by *post hoc* Bonferroni's multiple comparison tests between the groups.

## Conflict of interest

The authors declare no conflict of interest.

## Author contributions

RI, NN, and SM performed the experiments. SK and TI provided NCX^+/−^ mice and NCX3 antibody. SK, TI, and KF provided critical advice. RI and SM designed the study and prepared the manuscript.

### Peer review

The peer review history for this article is available at https://www.webofscience.com/api/gateway/wos/peer‐review/10.1111/febs.17339.

## Data Availability

All data are provided in the main text.

## References

[1] Faraone SV , Asherson P , Banaschewski T , Biederman J , Buitelaar JK , Ramos‐Quiroga JA , Rohde LA , Sonuga‐Barke EJS , Tannock R & Franke B (2015) Attention‐deficit/hyperactivity disorder. Nat Rev Dis Primers 1, 15020. 27189265 10.1038/nrdp.2015.20

[2] Gantz SC , Ford CP , Morikawa H & Williams JT (2018) The evolving understanding of dopamine neurons in the substantia nigra and ventral tegmental area. Annu Rev Physiol 80, 219–241. 28938084 10.1146/annurev-physiol-021317-121615

[3] Ernst M , Zametkin AJ , Matochik JA , Jons PH & Cohen RM (1998) DOPA decarboxylase activity in attention deficit hyperactivity disorder adults. A [fluorine‐18]fluorodopa positron emission tomographic study. J Neurosci 18, 5901–5907. 9671677 10.1523/JNEUROSCI.18-15-05901.1998 PMC6793062

[4] Arnsten AFT & Li B‐M (2005) Neurobiology of executive functions: catecholamine influences on prefrontal cortical function. Biol Psychiatry 57, 1377–1384. 15950011 10.1016/j.biopsych.2004.08.019

[5] DiMaio S , Grizenko N & Joober R (2003) Dopamine genes and attention‐deficit hyperactivity disorder: a review. J Psychiatry Neurosci 28, 27–38. 12587848 PMC161723

[6] Baker PF & McNaughton PA (1976) Kinetics and energetics of calcium efflux from intact squid giant axons. J Physiol 259, 103–144. 182956 10.1113/jphysiol.1976.sp011457 PMC1309017

[7] Blaustein MP & Santiago EM (1977) Effects of internal and external cations and of ATP on sodium‐calcium and calcium‐calcium exchange in squid axons. Biophys J 20, 79–111. 901903 10.1016/S0006-3495(77)85538-0 PMC1473341

[8] Li Z , Matsuoka S , Hryshko LV , Nicoll DA , Bersohn MM , Burke EP , Lifton RP & Philipson KD (1994) Cloning of the NCX2 isoform of the plasma membrane Na(+)‐Ca^2+^ exchanger. J Biol Chem 269, 17434–17439. 8021246

[9] Nicoll DA , Quednau BD , Qui Z , Xia YR , Lusis AJ & Philipson KD (1996) Cloning of a third mammalian Na^+^‐Ca^2+^ exchanger, NCX3. J Biol Chem 271, 24914–24921. 8798769 10.1074/jbc.271.40.24914

[10] Matsuda T , Takuma K , Nishiguchi E , Hashimoto H , Azuma J & Baba A (1996) Involvement of Na^+^–Ca^2+^ exchanger in reperfusion‐induced delayed cell death of cultured rat astrocytes. Eur J Neurosci 8, 951–958. 8743743 10.1111/j.1460-9568.1996.tb01582.x

[11] Atherton J , Kurbatskaya K , Bondulich M , Croft CL , Garwood CJ , Chhabra R , Wray S , Jeromin A , Hanger DP & Noble W (2014) Calpain cleavage and inactivation of the sodium calcium exchanger‐3 occur downstream of Aβ in Alzheimer's disease. Aging Cell 13, 49–59. 23919677 10.1111/acel.12148 PMC4326873

[12] Moriguchi S , Kita S , Fukaya M , Osanai M , Inagaki R , Sasaki Y , Izumi H , Horie K , Takeda J , Saito T *et al*. (2018) Reduced expression of Na^+^/Ca^2+^ exchangers is associated with cognitive deficits seen in Alzheimer's disease model mice. Neuropharmacology 131, 291–303. 29274751 10.1016/j.neuropharm.2017.12.037

[13] Sirabella R , Sisalli MJ , Costa G , Omura K , Ianniello G , Pinna A , Morelli M , Renzo GMD , Annunziato L & Scorziello A (2018) NCX1 and NCX3 as potential factors contributing to neurodegeneration and neuroinflammation in the A53T transgenic mouse model of Parkinson's disease. Cell Death Dis 9, 725. 29941946 10.1038/s41419-018-0775-7 PMC6018508

[14] XiaoCan J , YongLi Y , YuanCheng C , ZhiWei C , Yuhui D , Zhenhua X , Weiping Z , Chao X , Qiang Z , Xin X *et al*. (2019) Multivariate analysis of genome‐wide data to identify potential pleiotropic genes for five major psychiatric disorders using MetaCCA. J Affect Disord 242, 234–243. 30212762 10.1016/j.jad.2018.07.046 PMC6343670

[15] Mazei‐Robison MS , Couch RS , Shelton RC , Stein MA & Blakely RD (2005) Sequence variation in the human dopamine transporter gene in children with attention deficit hyperactivity disorder. Neuropharmacology 49, 724–736. 16171832 10.1016/j.neuropharm.2005.08.003

[16] Mazei‐Robison MS , Bowton E , Holy M , Schmudermaier M , Freissmuth M , Sitte HH , Galli A & Blakely RD (2008) Anomalous dopamine release associated with a human dopamine transporter coding variant. J Neurosci 28, 7040–7046. 18614672 10.1523/JNEUROSCI.0473-08.2008 PMC2573963

[17] Sakrikar D , Mazei‐Robison MS , Mergy MA , Richtand NW , Han Q , Hamilton PJ , Bowton E , Galli A , Veenstra‐Vanderweele J , Gill M *et al*. (2012) Attention deficit/hyperactivity disorder‐derived coding variation in the dopamine transporter disrupts microdomain targeting and trafficking regulation. J Neurosci 32, 5385–5397. 22514303 10.1523/JNEUROSCI.6033-11.2012 PMC3342037

[18] Mergy MA , Gowrishankar R , Gresch PJ , Gantz SC , Williams J , Davis GL , Wheeler CA , Stanwood GD , Hahn MK & Blakely RD (2014) The rare DAT coding variant Val559 perturbs da neuron function, changes behavior, and alters in vivo responses to psychostimulants. Proc Natl Acad Sci USA 111, E4779–E4788. 25331903 10.1073/pnas.1417294111 PMC4226116

[19] Bowton E , Saunders C , Erreger K , Sakrikar D , Matthies HJ , Sen N , Jessen T , Colbran RJ , Caron MG , Javitch JA *et al*. (2010) Dysregulation of dopamine transporters via dopamine D2 autoreceptors triggers anomalous dopamine efflux associated with attention‐deficit hyperactivity disorder. J Neurosci 30, 6048–6057. 20427663 10.1523/JNEUROSCI.5094-09.2010 PMC2881830

[20] Keighron JD , Jbonaventura J , Li Y , Yang JW , DeMarco EM , Hersey M , Cao J , Sandtner W , Michaelides M , Sitte HH *et al*. (2023) Interactions of calmodulin kinase II with the dopamine transporter facilitate cocaine‐induced enhancement of evoked dopamine release. Transl Psychiatry 13, 202. 37311803 10.1038/s41398-023-02493-4 PMC10264427

[21] Fog JU , Khoshbouei H , Holy M , Owens WA , Vaegter CB , Sen N , Nikandrova Y , Bowton E , McMahon DG , Colbran RJ *et al*. (2006) Calmodulin kinase II interacts with the dopamine transporter C terminus to regulate amphetamine‐induced reverse transport. Neuron 51, 417–429. 16908408 10.1016/j.neuron.2006.06.028

[22] Dauer W & Przedborski S (2003) Parkinson's disease: mechanisms and models. Neuron 39, 889–909. 12971891 10.1016/s0896-6273(03)00568-3

[23] Otani S , Daniel H , Roisin MP & Crrepel F (2003) Dopaminergic modulation of long‐term synaptic plasticity in rat prefrontal neurons. Cereb Cortex 13, 1251–1256. 14576216 10.1093/cercor/bhg092

[24] Sotoyama H , Inaba H , Iwakura Y , Namba H , Takei N , Sasaoka T & Nawa H (2022) The dual role of dopamine in the modulation of information processing in the prefrontal cortex underlying social behavior. FASEB J 36, e22160. 35064699 10.1096/fj.202101637R

[25] West AE , Griffith EC & Greenberg ME (2002) Regulation of transcription factors by neuronal activity. Nat Rev Neurosci 3, 921–931. 12461549 10.1038/nrn987

[26] Boekhoudt L , Omrani A , Luijendijk MCM , Wolterink‐Donselaar IG , Wijbrans EC , van der Plasse G & Adan RAH (2016) Chemogenetic activation of dopamine neurons in the ventral tegmental area, but not substantia nigra, induces hyperactivity in rats. Eur Neuropsychopharmacol 26, 1784–1793. 27712862 10.1016/j.euroneuro.2016.09.003

[27] Williams GV & Goldman‐Rakic PS (1995) Modulation of memory fields by dopamine D1 receptors in prefrontal cortex. Nature 376, 572–575. 7637804 10.1038/376572a0

[28] Wang M , Vijayraghavan S & Goldman‐Rakic PS (2004) Selective D2 receptor actions on the functional circuitry of working memory. Science 303, 853–856. 14764884 10.1126/science.1091162

[29] Tritsch NX & Sabatini BL (2012) Dopaminergic modulation of synaptic transmission in cortex and striatum. Neuron 76, 33–50. 23040805 10.1016/j.neuron.2012.09.023 PMC4386589

[30] Seong HJ & Carter AG (2012) D1 receptor modulation of action potential firing in a subpopulation of layer 5 pyramidal neurons in the prefrontal cortex. J Neurosci 32, 10516–10521. 22855801 10.1523/JNEUROSCI.1367-12.2012 PMC3429120

[31] Xing B , Mack NR , Guo KM , Zhang YX , Ramirez B , Yang SS , Lin L , Wang DV , Li YC & Gao WJ (2021) A subpopulation of prefrontal cortical neurons is required for social memory. Biol Psychiatry 89, 521–531. 33190846 10.1016/j.biopsych.2020.08.023 PMC7867585

[32] Xing B , Mack NR , Zhang YX , McEachern EP & Gao WJ (2022) Distinct roles for prefrontal dopamine D1 and D2 neurons in social hierarchy. J Neurosci 42, 313–324. 34844989 10.1523/JNEUROSCI.0741-21.2021 PMC8802944

[33] Murphy BL , Arnsten ATF , Goldman‐Rakic PS & Roth RH (1996) Increased dopamine turnover in the prefrontal cortex impairs spatial working memory performance in rats and monkeys. Proc Natl Acad Sci USA 93, 1325–1329. 8577763 10.1073/pnas.93.3.1325 PMC40079

[34] Zahrt J , Taylor JR , Mathew RG & Arnsten AFT (1997) Supranormal stimulation of D1 dopamine receptors in the rodent prefrontal cortex impairs spatial working memory performance. J Neurosci 17, 8528–8535. 9334425 10.1523/JNEUROSCI.17-21-08528.1997 PMC6573725

[35] Beaulieu JM , Sotnikova TD , Gainetdinov RR & Caron MG (2006) Paradoxical striatal cellular signaling responses to psychostimulants in hyperactive mice. J Biol Chem 281, 32072–32080. 16954211 10.1074/jbc.M606062200

[36] Xu TX , Sotnikova TD , Liang C , Zhang J , Jung JU , Spealman RD , Gainetdinov RR & Yao WD (2009) Hyperdopaminergic tone erodes prefrontal long‐term potential via a D2 receptor‐operated protein phosphatase gate. J Neurosci 29, 14086–14099. 19906957 10.1523/JNEUROSCI.0974-09.2009 PMC2818669

[37] Leo D , Sukhanov I , Zoratto F , Illiano P , Caffino L , Sanna F , Messa G , Emanuele M , Esposito A , Dorofeikova M *et al*. (2018) Pronounced hyperactivity, cognitive dysfunctions, and BDNF dysregulation in dopamine transporter knock‐out rats. J Neurosci 38, 1959–1972. 29348190 10.1523/JNEUROSCI.1931-17.2018 PMC5824739

[38] Steinkellner T , Mus L , Eisenrauch B , Constantinescu A , Leo D , Konrad L , Rickhag M , Sørensen G , Efimova EV , Kong E *et al*. (2014) In vivo amphetamine action is contingent on αCaMKII. Neuropsychopharmacology 39, 2681–2693. 24871545 10.1038/npp.2014.124 PMC4207348

[39] Schlman H & Lou LL (1989) Multifunctional Ca^2+^/calmodulin‐dependent protein kinase: domain structure and regulation. Trends Biochem Sci 14, 62–66. 2539662 10.1016/0968-0004(89)90045-5

[40] Klee CB (1991) Concerted regulation of protein phosphorylation and dephosphorylation by calmodulin. Neurochem Res 16, 1059–1065. 1664495 10.1007/BF00965851

[41] Bano D , Young KW , Guerin CJ , Lefeuvre R , Rothwell NJ , Naldini L , Rizzuto R , Carafoli E & Nicotera P (2005) Cleavage of the plasma membrane Na^+^/Ca^2+^ exchanger in excitotoxicity. Cell 120, 275–285. 15680332 10.1016/j.cell.2004.11.049

[42] Altamirano F , Eltit JM , Robin G , Linares N , Ding X , Pessah IN , Allen PD & López JR (2014) Ca^2+^ influx via the Na^+^/Ca^2+^ exchanger is enhanced in malignant hyperthermia skeletal muscle. J Biol Chem 289, 19180–19190. 24847052 10.1074/jbc.M114.550764 PMC4081953

[43] Giese KP , Fedorov NB , Filipkowski RK & Silva AJ (1998) Autophosphorylation at Thr286 of the alpha calcium‐calmodulin kinase II in LTP and learning. Science 279, 870–873. 9452388 10.1126/science.279.5352.870

[44] Elgersma Y , Fedorov NB , Ikonen S , Choi ES , Elgersma M , Carvalho OM , Giese KP & Silva AJ (2002) Inhibitory autophosphorylation of CaMKII controls PSD association, plasticity, and learning. Neuron 36, 493–505. 12408851 10.1016/s0896-6273(02)01007-3

[45] Weeber EJ , Jiang YH , Elgersma Y , Varga AW , Carrasquillo Y , Brown SE , Christian JM , Mirinikjoo B , Silva A , Beaudet AL *et al*. (2003) Derangements of hippocampal calcium/calmodulin‐dependent protein kinase II in a mouse model for Angelman mental retardation syndrome. J Neurosci 23, 2634–2644. 12684449 10.1523/JNEUROSCI.23-07-02634.2003 PMC6742065

[46] Jiang YH , Pan Y , Zhu L , Landa L , Yoo J , Spencer C , Lorenzo I , Brilliant M , Noebels J & Beaudet AL (2010) Altered ultrasonic vocalization and impaired learning and memory in Angelman syndrome mouse model with a large maternal deletion from Ube3a to Gabrb3. PLoS One 5, e12278. 20808828 10.1371/journal.pone.0012278 PMC2924885

[47] Nogami T , Beppu H , Tokoro T , Moriguchi S , Shioda N , Fukunaga K , Ohtsuka T , Ishii Y , Sasahara M , Shimada Y *et al*. (2011) Reduced expression of the ATRX gene, a chromatin‐remodeling factor, causes hippocampal dysfunction in mice. Hippocampus 21, 678–687. 20865721 10.1002/hipo.20782

[48] Shioda N , Beppu H , Fukuda T , Li E , Kitajima I & Fukunaga K (2011) Aberrant calcium/calmodulin‐dependent protein kinase II (CaMKII) activity is associated with abnormal dendritic spine morphology in the ATRX mutant mouse brain. J Neurosci 31, 346–358. 21209221 10.1523/JNEUROSCI.4816-10.2011 PMC6622766

[49] Yabuki Y , Shioda N , Maeda T , Hiraide S , Togashi H & Fukunaga K (2014) Aberrant CaMKII activity in the medial prefrontal cortex is associated with cognitive dysfunction in ADHD model rats. Brain Res 1557, 90–100. 24561222 10.1016/j.brainres.2014.02.025

[50] Chi P , Greengard P & Ryan TA (2001) Synapsin dispersion and reclustering during synaptic activity. Nat Neurosci 4, 1187–1193. 11685225 10.1038/nn756

[51] Pothos EN , Przedborski S , Davila V , Schmitz Y & Sulzer D (1998) D2‐like dopamine autoreceptor activation reduces quantal size in PC12 cells. J Neurosci 18, 5575–5585. 9671649 10.1523/JNEUROSCI.18-15-05575.1998 PMC6793067

[52] Fan X , Xu M & Hess EJ (2010) D2 dopamine receptor subtype‐mediated hyperactivity and amphetamine responses in a model of ADHD. Neurobiol Dis 37, 228–236. 19840852 10.1016/j.nbd.2009.10.009 PMC2839459

[53] Arnsten AFT & Dudley AG (2005) Methylphenidate improves prefrontal cortical cognitive function through α2 adrenoceptor and dopamine D1 receptor actions: relevance to therapeutic effects in attention deficit hyperactivity disorder. Behav Brain Funct 1, 2. 15916700 10.1186/1744-9081-1-2 PMC1143775

[54] Liu XY , Chu XP , Mao LM , Wang M , Lan HX , Li MH , Zhang GC , Parelkar NK , Fibuch EE , Haines M *et al*. (2006) Modulation of D2R‐NR2B interactions in response to cocaine. Neuron 52, 897–909. 17145509 10.1016/j.neuron.2006.10.011

[55] Svenningsson P , Nishi A , Fisone G , Girault JA , Nairn AC & Greengard P (2004) DARPP‐32: an integrator of neurotransmission. Annu Rev Pharmacol Toxicol 44, 269–296. 14744247 10.1146/annurev.pharmtox.44.101802.121415

[56] Bateup HS , Svenningsson P , Kuroiwa M , Gong S , Nishi A , Heintz N & Greengard P (2008) Cell type‐specific regulation of DARPP‐32 phosphorylation by psychostimulant and antipsychotic drugs. Nat Neurosci 11, 932–939. 18622401 10.1038/nn.2153 PMC2737705

[57] Scorziello A , Savoia C , Sisalli MJ , Adornetto A , Secondo A , Boscia F , Esposito A , Polishchuk EV , Polishchuk RS , Molinaro P *et al*. (2013) NCX3 regulates mitochondrial Ca (2+) handling through the AKAP121‐anchored signaling complex and prevents hypoxia‐induced neuronal death. J Cell Sci 126, 5566–5577. 24101730 10.1242/jcs.129668

[58] Wakimoto K , Fujimura H , Iwamoto T , Oka T , Kobayashi K , Kita S , Kudoh S , Kuro‐o M , Nabeshima Y , Shigekawa M *et al*. (2003) Na^+^/Ca^2+^ exchanger‐deficient mice have disorganized myofibrils and swollen mitochondria in cardiomyocytes. Comp Biochem Physiol B Biochem Mol Biol 135, 9–15. 12781968 10.1016/s1096-4959(03)00057-5

[59] Kon N , Wang HT , Kato YS , Uemoto K , Kawamoto N , Kawasaki K , Enoki R , Kurosawa G , Nakane T , Sugiyama Y *et al*. (2021) Na^+^/Ca^2+^ exchanger mediates cold Ca^2+^ signaling conserved for temperature‐compensated circadian rhythms. Sci Adv 7, eabe8132. 33931447 10.1126/sciadv.abe8132 PMC8087402

[60] Gotoh Y , Kita S , Fujii M , Tagashira H , Horie I , Arai Y , Uchida S & Iwamoto T (2015) Genetic knockout and pharmacologic inhibition of NCX2 cause natriuresis and hypercalciuria. Biochem Biophys Res Commun 456, 670–675. 25498502 10.1016/j.bbrc.2014.12.016

[61] Islam MR , Moriguchi S , Tagashira H & Fukunaga K (2014) Rivastigmine improves hippocampal neurogenesis and depression‐like behaviors via 5‐HT1A receptor stimulation in olfactory bulbectomized mice. Neuroscience 2729, 116–130. 10.1016/j.neuroscience.2014.04.046 24797332

[62] Moriguchi S , Inagaki R , Saito T , Saido TC & Fukunaga K (2022) Propolis promotes memantine‐dependent rescue of cognitive deficits in APP‐KI mice. Mol Neurobiol 59, 4630–4646. 35587310 10.1007/s12035-022-02876-6

[63] Moriguchi S , Inagaki R & Fukunaga K (2021) Memantine improves cognitive deficits via KATP channel inhibition in olfactory bulbectomized mice. Mol Cell Neurosci 117, 103680. 34715352 10.1016/j.mcn.2021.103680

[64] Inagaki R , Moriguchi S & Fukunaga K (2018) Aberrant amygdala‐dependent fear memory in corticosterone‐treated mice. Neuroscience 388, 448–459. 30118751 10.1016/j.neuroscience.2018.08.004

[65] Iwamoto T , Pan Y , Nakamura TY , Wakabayashi S & Shigekawa M (1998) Protein kinase C‐dependent regulation of Na^+^/Ca^2+^ exchanger isoforms NCX1 and NCX3 does not require their direct phosphorylation. Biochemistry 37, 17230–17238. 9860837 10.1021/bi981521q

[66] Wang X , Hatatani K , Sun Y , Fukumachi T , Saito H & Kobayashi H (2012) TCR signaling via ZAP‐70 induced by CD3 stimulation is more active under acidic conditions. J Cell Sci Ther 15, 002.

[67] Inagaki R , Yamakuni T , Saito T , Saido TC & Moriguchi S (2023) Preventive effect of propolis on cognitive decline in Alzheimer's disease model mice. Neurobiol Aging 139, 20–29. 10.1016/j.neurobiolaging.2024.03.002 38583392

[68] Inagaki R , Moriguchi S & Fukunaga K (2020) Kir6.1 heterozygous mice exhibit aberrant amygdala‐dependent cued fear memory. Mol Neurobiol 57, 1622–1635. 31808063 10.1007/s12035-019-01840-1

[69] Nitta A , Izuo N , Hamatani K , Inagaki R , Kusui Y , Fu K , Asano T , Torii Y , Habuchi C , Sekiguchi H *et al*. (2021) Schizophrenia‐like behavioral impairments in mice with suppressed expression of piccolo in the medial prefrontal cortex. J Pers Med 11, 607. 34206873 10.3390/jpm11070607 PMC8304324

